# Paramedic Roles, Purpose, and Practices When Responding to Older Adults in Abusive Contexts: A Systematic Review

**DOI:** 10.1177/07334648251330347

**Published:** 2025-04-02

**Authors:** Megan De Silva, Aidan Peters, Benjamin De Waal, Lloyd Christopher, Navindhra Naidoo

**Affiliations:** 1 Paramedicine, School of Health Sciences, 67422 Western Sydney University, Penrith, NSW, Australia; 2 Faculty of Health and Wellness Sciences, 70683 Cape Peninsula University of Technology, Cape Town, South Africa; 3 NSW Ambulance, Sydney Olympic Park, NSW, Australia

**Keywords:** abuse and neglect, health disparities, health outcomes, quality of life, systematic review

## Abstract

Older adults are disproportionately exposed to increased harm and adverse outcomes in abusive contexts. This systematic review aimed to explore and evaluate current literature on the role of paramedics responding to older adults in abusive contexts, with a specific focus on the vulnerability-reducing and protective potential of paramedics. A systematic review of the literature was undertaken using the Joanna Briggs Institute Systematic Review Guidelines. From an initial yield of 584 results, 18 sources of evidence met the criteria for inclusion and subsequent data extraction. Predominantly North-American literature indicated that older adults experience nuanced, disproportionate and complex vulnerability in abusive contexts, and that paramedics have a poorly defined and inconsistently applied duty of care. Consequently, this systematic review provides key recommendations for enhancing the capacity of paramedics to respond to the unmet needs and vulnerabilities of older adults either experiencing or at risk of abuse or neglect.

**What this paper adds**

• Older adults experience nuanced, disproportionate and complex vulnerability in abusive contexts. This paper highlights the critical role of paramedics in identifying, responding to, and reporting abuse and neglect of older adults, emphasising their unique position as first responders who access vulnerable populations in their homes.
• Through systematically reviewing 18 sources of evidence, the paper identified persistent barriers to reporting, whilst providing recommendations to significantly increase reporting rates. It also advocates for interprofessional collaboration among paramedics, emergency department staff, and social services to improve longitudinal care and outcomes for older adults experiencing abuse or neglect.

**Application of study findings**

• The paper recommends the development and integration of evidence-informed prehospital screening tools and protocols for detecting and reporting abuse of older adults, which could enhance paramedics' ability to effectively address abuse of older adults. It also supports the implementation of mandatory, evidence-based paramedic training programs focused on recognizing and responding to abuse of older adults, including jurisdiction-specific reporting guidelines.
• The paper calls for strengthened partnerships between paramedics, emergency department staff and social services to improve the continuum of care for older adults, with a potential for leveraging community paramedicine to provide ongoing support and monitoring for at risk individuals.

## Introduction

Abusive contexts refer to conditions that undermine one’s autonomy and threaten physical, psychological or economic safety and security (Australian Institute of Health and Welfare, 2019). This could include domestic and other interpersonal violence, neglect, exploitation or deprivation of care or resources (relative to need) (Bond & Butler, 2013). For older adults, exposure to such social determinants of health is often serial and protracted in nature, nuanced when the people who use violence or other forms of abuse are family members or those charged with the noble responsibility of care.

It is estimated that approximately 5–10% of all older adults experience mistreatment each year (Rosen et al., 2017), with consequences that include severely increased rates of morbidity and mortality (Mercier et al., 2020). This mistreatment or abuse can include physical abuse, sexual abuse or sexually abusive contact, emotional or psychological abuse, financial abuse or exploitation, or neglect (Nowak et al., 2018; Rosen et al., 2017). Risk of abuse is further enhanced before, during and after large scale crises (Whiteman et al., 2023). While cases of abuse of older adults, neglect, and self-neglect are common in geriatric practice, and may have serious medical consequences, they are rarely identified and are underreported in out-of-hospital settings (Mercier et al., 2020; Rosen et al., 2017). Social isolation, dementia, health and functional status, are risk factors for abuse and may prevent older adults from identifying and self-reporting abuse or neglectful situations (Cannell et al., 2016; Gonzalez et al., 2016). Older people living with cognitive or functional limitations may also fear retaliation from family members or caregivers and continue to live in abusive situations (Gonzalez et al., 2016), with levels of dependence on caregivers influencing the risk of an older person being physically abused or neglected (Mercier et al., 2020). With specific regard to physical findings, two-thirds of injuries sustained secondary to abuse of older adults were located on the upper extremities and maxillofacial region (Mercier et al., 2020). Literature also identified females are being more commonly subjected to neglect, physical, and psychological abuse, often due to discriminatory societal attitudes and non-realization of their human rights (Mercier et al., 2020; United Nations [UN], 2002).

Abuse of older adults is associated with long-term debilitating psychological effects, including anxiety, depression, and suicidal ideation, as well as increased risk of hospitalization, long-term facility placement, and death (Mercier et al., 2020; Rosen et al., 2017). This is in addition to “self-neglect,” with some older adults threatening their own health and safety by failing or refusing to seek assistance with essential self-care (Rosen et al., 2017). The impact of the trauma caused by abuse, or self-neglect, may be worsened by shame and fear, causing reluctance to seek help (UN, 2002). Consequently, delays in recognizing abuse have detrimental effects on the older person’s quality of life and health outcomes (Mercier et al., 2020). As the aging process decreases rates of healing, some older victim-survivors *never* recover physically or emotionally from the trauma inflicted by the abuse (UN, 2002). Older adults who experience abuse or neglect also have significantly increased mortality rates than those who lack exposure to abuse (Rosen et al., 2017).

Considered an emerging profession due to undergoing significant transitions in recent decades, including the establishment of tertiary-based education as a prerequisite for entry into the field, and national registration in countries including Australia, New Zealand and Canada (Naidoo, Maria, et al., 2024), the role of paramedics as front-line responders to abuse of older adults lacks clear articulation. Despite their unique societal positionality and inherent professional capability to assess and respond to medical, trauma, social, and psychological emergencies across the lifespan, their capacity and scope of responsibilities within abusive contexts remains undefined.

Within the scope of this study, the authors take an inclusive approach to defining paramedics, including emergency medical technicians (EMT), emergency medical services (EMS), and prehospital primary care and advanced care practitioners. While each of these professionals may lack homogeneity in scope, jurisdictional guidelines and responsibilities, all operate in out-of-hospital emergency contexts (including aged care or residential facilities) and have the same fundamental duty of care. As the only healthcare providers who routinely enter patient’s homes, and often as the first medically trained personnel to evaluate ill and injured older patients, paramedics are uniquely positioned to recognize and respond to signs of abuse. By exploring the experiences, perceptions, and challenges faced by paramedics in responding to the abuse of older adults, this systematic review aims to explore and evaluate the published and gray literature on the paramedics’ role and capacity to reduce vulnerability among older adults residing within abusive contexts, inclusive of, but not limited to, physical, sexual, emotional/psychological, and financial abuse, and neglect. By synthesizing existing literature, the authors seek to highlight the evidenced role of paramedics within contexts of abuse, neglect or deprivation and make policy and practice recommendations to enhance the effectiveness of paramedic response.

## Methods

A systematic review of the literature was undertaken using the PRISMA 2020 framework (Page et al., 2021) and the Joanna Briggs Institute (JBI) guidelines (JBI, 2024). Four databases (Medline via PubMed, CINAHL, the Cochrane Library of Systematic Reviews, and JBI Evidence Based Practice) were searched with the PRISMA searching extension (Rethlefsen et al., 2021) on 18 March 2024, with results replicated on 20 March 2024 and 15 August 2024 for recency. Searches were limited to full-text availability and publications after 2000, in peer-reviewed English language journals, to ensure inclusion of contemporary practices and policies, and methodologically robust research. Gray literature from the United Nations (UN, nd), the World Health Organization (WHO) (WHO, nd), and the National Ageing Research Institute (National Ageing Research Institute, nd) were hand-searched on 21 March 2024 and 15 August 2024 to increase the breadth of evidence analyzed, and reduce potential publication or selection bias (Paez, 2017). Hand-searching, defined as manual page-by-page examination of relevant databases, journals, studies and conference proceedings (identified in the reference lists of accepted articles), was employed to ensure comprehensive identification of all eligible evidence (Lefebvre et al., 2021). The same title and abstract eligibility criteria were applied to sources derived from the hand search.

Database specific search strings were peer-reviewed by an information scientist and are included in Appendix 1. An a priori review protocol was registered on Open Science Framework (OSF) (https://osf.io/nkeh7/). In the absence of human participants, the study meets the threshold for ethics exemption.

### Study Selection

Results from the database searches were appraised by title and abstract by MD and AP against the pre-defined inclusion criteria using the Covidence™ software (Veritas Health Innovation, 2024) between the 16 and 19August 2024. Inclusion criteria were established through the population, concept, and context acronym by MD and NN, as recommended by JBI (JBI, 2024) (Table 1). Studies that examined the role of paramedics (including paramedic students, graduates, first responders, EMS and EMT personnel) in identification and response to abusive contexts among adults aged 65 years and older, were eligible for inclusion. Full-text articles retained after title and abstract screening were reviewed with disputes resolved via consensus-finding between all authors. Reference lists of articles eligible for inclusion following full-text screening were hand searched on 8 October 2024 by LC with an additional 33 potentially relevant articles identified. Following the removal of 24 duplicates, 9 articles underwent additional title and abstract screening by MD and NN, with 2 articles progressing to full-text screening. Fourteen documents identified during initial hand searches of gray literature were screened in full during full-text screening by MD and AP, due to the unavailability of abstracts. Following full-text screening 18 sources of evidence met the inclusion criteria and were advanced for data extraction and synthesis. Full exclusion criteria are listed in Table 2.

**Table 1.** PCC Development.

Population	Paramedics, paramedic students, paramedic graduates, first responders, emergency medical services (EMS), emergency medical technicians (EMTs), older adults (defined as people at or over the age of 65)
Concept	Paramedic identification and response to vulnerabilities
Context	Abusive contexts including, but not limited to, institutional abuse, parental abuse, or gerontologic abuse.

**Table 2.** Exclusion Criteria.

Wrong population	- Animal studies
	- Any study that does not include paramedics, paramedic students, paramedic graduates, first responders, EMS, EMTs
	- Any study without older adults (65 years and older)
Wrong context	- In-hospital studies
	- Any study that does not include abusive contexts
Wrong methodology	- Any study published before 2000
	- Any study not published in English (language)
	- Any study that does not have available full-text paper/s
Wrong outcome	- Any study that does not include assessment of risk, determination of need, or specific professional response

### Data Extraction and Synthesis

Data extraction was completed independently by MD and NN using the Covidence™ software (Veritas Health Innovation, 2024). All studies that met inclusion criteria underwent data extraction for author(s), country and year of publication, context, study type, study objective (or aim), key findings, and recommendations or professional response. Consensus on the precision of data extracted was reached via discussion between MD and NN before the synthesis of evidence using Microsoft Word™ and Excel™.

### Quality Appraisal

Quality appraisal was conducted to ensure the credibility, reliability and validity of included studies, therefore allowing transparent assessment of potential biases (Muka et al., 2020). Quality appraisal was performed with the JBI analytical appraisal tools for textual evidence: policy (McArthur et al., 2020), diagnostic test accuracy studies (Campbell JM, 2020), qualitative research (Lockwood C, 2015), case series (Munn, Aromataris, et al., 2020), quasi-experimental studies (Barker et al., 2024), expert opinion (McArthur et al., 2020), and prevalence studies (Munn et al., 2020) respectively, by AP and BD. The quality appraisal tools consisted of six to ten criteria, dependent on study type, with studies receiving a “yes” response to half or more of the domains considered to have a low level of bias, or a greater than 60% methodological quality rating (Dahoud et al., 2024). All included articles (n = 18) were considered to have a low level of bias and remained eligible for inclusion (Appendix 2).

### Certainty of Evidence Assessment

Certainty of evidence was undertaken by BD, LC and NN for each of the study’s findings using a variety of software/s specific to study type to further ensure the reliability of results and strengthen the reported findings (Muka et al., 2020). BD and NN employed GRADEpro (2024) software for quantitative reviews while LC and NN employed GRADE CerQUal (nd) for qualitative reviews. One study (Mercier et al., 2020) did not undergo certainty of evidence assessment due to the unavailability of validated certainty of evidence tools or software for scoping reviews. The authors acknowledge that the absence of formal assessment may decrease the strength and reliability of included findings, due to the potential introduction of selection and interpretation bias influencing the overall conclusions drawn from the review. Full certainty of evidence assessment results can be found in Appendix 3.

## Results

### Search Results

**Figure 1.**
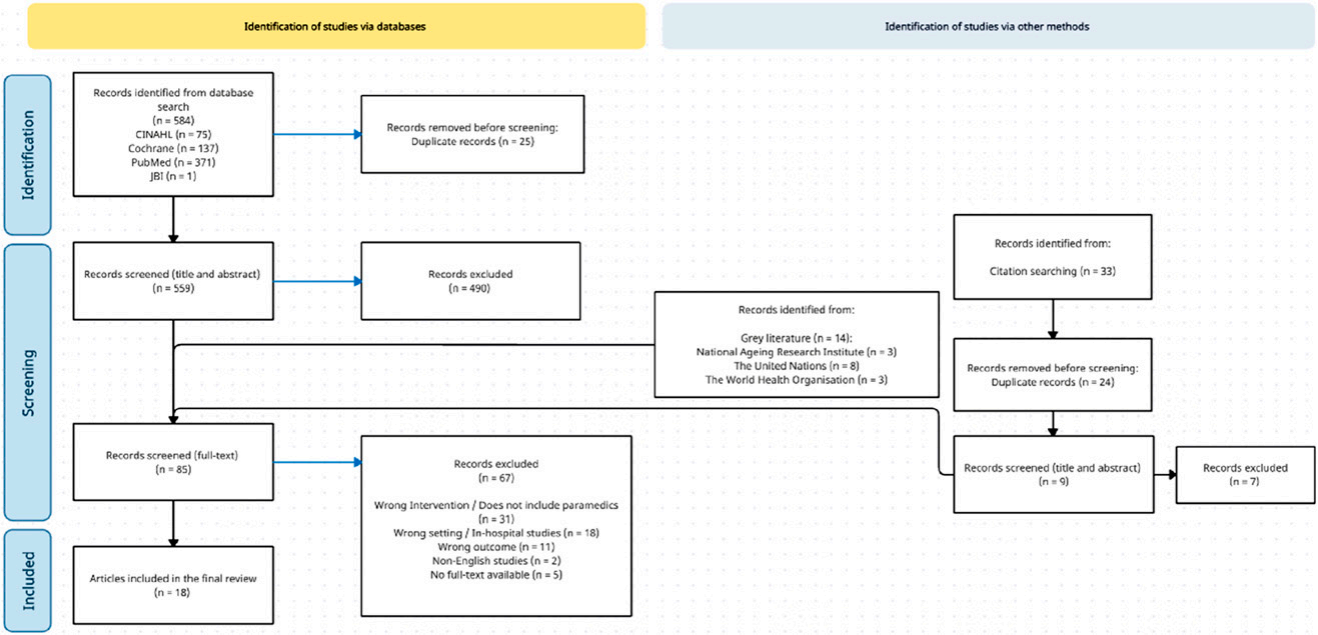
PRISMA diagram.

The database literature search produced 584 results, with the screening and inclusion process illustrated in Figure 1 (PRISMA Flow Diagram). Twenty-five duplicate articles were removed. The remaining 559 articles underwent independent title and abstract screening, with 490 studies excluded due to not meeting inclusion criteria. A total of 83 studies underwent full-text screening, which included fourteen sources of evidence identified during hand searches of gray literature and were included in the full-text screening stage. Sixteen sources of evidence were eligible for data extraction and inclusion. From these 16 sources, an additional 33 potentially relevant sources were identified during the hand search of reference lists (from the 16 sources). Following the removal of 24 duplicates, 9 articles identified during the hand search of reference lists underwent screening, with 7 articles removed at the title and abstract stage, and 2 progressing to data extraction following full-text screening. The final 18 articles/documents were considered eligible for inclusion.

### Characteristics of Included Studies

Of the 18 studies included for analysis, the majority were conducted in North America (*n* = 16) (Cannell et al., 2016, 2019; Cannell, Livingston, et al., 2020; Cannell, Mars, & Schoen, 2020; Cimino-Fiallos & Rosen, 2021; Friese & Collopy, 2010; Gironda et al., 2010; Gonzalez et al., 2016; Kue et al., 2009; Mercier et al., 2020; Namboodri et al., 2018; Nowak et al., 2018; Nusbaum et al., 2006; Rinker, 2009; Rosen et al., 2017, 2018), with one study performed in Spain (UN, 2002), and one in Finland (Salminen-Tuomaala et al., 2021). Of these studies, four were qualitative studies (Cannell et al., 2016; Gonzalez et al., 2016; Rosen et al., 2017; Salminen-Tuomaala et al., 2021), while two were expert opinion (Cannell, Mars, & Schoen, 2020; Friese & Collopy, 2010), and three were text and opinion (Cimino-Fiallos & Rosen, 2021; Gironda et al., 2010; Rosen et al., 2018). Six quantitative studies included a survey (*n* = 1) (Nusbaum et al., 2006), a case series (*n* = 1) (Kue et al., 2009), a cross-sectional study (*n* = 1) (Namboodri et al., 2018), a cohort study (*n* = 1) (Cannell et al., 2019), a prevalence study (*n* = 1) (Cannell, Livingston, et al., 2020), and a descriptive study (*n* = 1) (Rinker, 2009). The final three studies included gray literature and policy (*n* = 1) (United Nations, 2002; Nowak et al., 2018) and a scoping review (Mercier et al., 2020). Complete characteristics of included studies are listed in Appendixes 4.1.

### Excluded Studies

Of the total 67 documents excluded during full-text screening, 31 were excluded for not including paramedics working in the out-of-hospital environment (wrong intervention). Eighteen studies included the wrong setting, such as studies performed in hospital settings, including studies primarily set in the emergency department and not including out-of-hospital contexts (wrong context), while 11 studies were excluded for not including assessment of risk, determination of need, or a specific professional paramedic response (wrong outcome). Additionally, 2 studies were excluded due to not being published in the English language, to reduce the risk of bias associated with translation, and 5 studies were excluded due to the unavailability of full-text articles, both open and/or closed access.

### Summary of Findings (SOF)

#### Quantitative Analysis

Seven sources of evidence were included in quantitative analysis via the GRADEPRO software (2024) (Table 3) (Cannell et al., 2019; Cannell, Livingston, et al., 2020; Kue et al., 2009; Namboodri et al., 2018; Nowak et al., 2018; Nusbaum et al., 2006; Rinker, 2009). Of the GRADE certainty of evidence screening for quantitative evidence, all outcomes were rated as low or very low certainty due to limitations, including the use of convenience sampling, self-reported data, retrospective matching and low sample sizes. These limitations decreased the robustness of the findings and confidence in the generalizability of the results. However, all seven sources of evidence demonstrated high levels of importance to the research question by providing salient data and perspectives which inform understanding and guide this paper’s findings and recommendations and inform future policy. Full GRADE Pro certainty of evidence explanations is listed in Table 3 and Appendixes 3.1.

**Table 3.** GRADE Summary of Findings Table (Quantitative Analysis).

Self-reported exposure, screening and referral of suspected elder abuse cases by prehospital emergency care providers.			
Outcomes	Impact	№ of participants (studies)	Certainty of the evidence (GRADE)
EMS Referral Rate for Suspected Elderly Abuse	The referral rate from EMS for suspected elderly abuse ranges from 0.5% to 4% of all reported cases of elderly abuse.^i,j^	(2 non-randomized studies)	⨁◯◯◯
			Very low^a^
Effectiveness of screening tools in increasing suspected abuse reporting and rates	Implementation of the DETECT Screening tool increased the reporting rate by 3-fold (RR 3.03, CI 0.95: 2.06 - 4.46) in one study. In a second study the implementation of the same tool increased reporting more than two-fold (increased by 226%, p = 0.0056).^i,k^	(2 non-randomized studies)	⨁◯◯◯
			Very low^b^
Use of mandatory screening requirements for increasing screening rate	Changing from a system of voluntary screening to mandatory screening using a validation rule on ePCRs increased the screening rate from 5% (n = 25/549) to 86% (1222/1418).^k^	(1 non-randomized study)	⨁⨁◯◯
			Low^c^
Accuracy of Prehospital Screening Tools for Elderly Abuse	In one study screening accuracy using the DETECT tool was reported at 82% (95% CI: 77% to 87%) as verified by post screening case investigation. In another study (DETECT screening tool pilot study), the screening detection rate was 10% confirmed through matching screening records with subsequent case investigations.^i,k^	(2 non-randomized studies)	⨁◯◯◯
			Very low^d^
EMS Self-Reported Exposure to Elderly Abuse	Between 58% to 65% of survey respondents indicated that they had encountered at least one case of suspected abuse in the past 6 months. In one study 64.3% of participants reported encountering at least 1 suspected case in the past 12 months. One study (Nowak et al, 2018) estimated the mean number of reported cases at 1.7 ± 5.0 per month and 18.1 ± 35.6 over the course of a career.^l,m,n^	(3 non-randomized studies)	⨁◯◯◯
			Very low^e^
EMS self-reported screening practices, believes and attitudes towards screening	60.5% to 70% of participants indicated that they had not screened any patients for suspected elderly abuse in the past 12 or 6 months respectively. In one study, Rinker, 2009 89% of respondents indicated they were aware of their obligation to report, but 76% indicated that they did not believe that elderly abuse is a medical problem, with 20% indicating it is a social problem. In the same study 96.5% of participants indicated that they did not believe elderly abuse was a rare event.^m,n^	(2 non-randomized studies)	⨁◯◯◯
			Very low^e,f^
Formalized EMS Protocols for Elderly Abuse Screening and Reporting	40% (n = 14/35) of included states (USA) had formalized EMS protocols addressing elderly abuse. A total of 17.1% (n = 6/35) provide definitions for elderly abuse, 29% (n = 10/35) describe indicators for abuse, 22.8% (n = 8/35) provide guidelines for management and 34.3% (n = 12/35) provide instructions on reporting suspected abuse cases.^o^	(1 non-randomized study)	⨁◯◯◯
			Very low^g,h^

^a^For one study (Cannell, Livingston, et al., 2020) this data was reported but was not part of the objectives of the study.

^b^The study did not measure confirmed cases of abuse. It only matched screening reports to reports of investigations.

^c^This study used a quasi-experimental design.

^d^One of the studies (Cannell et al., 2019) was a pilot study. The authors retrospectively matched positive screening records with subsequent investigation records. As such there is no confirmation for false positive or false negative screening outcomes. As such the authors did not report on more formal measures of diagnostics accuracy.

^e^The included survey studies used convenience samples of participants and self-reported practice.

^f^One of the studies included both hospital-based and prehospital-based providers.

^g^This study only included publicly available protocols which could be found on public facing web interfaces.

^h^Sample size limitations, wide confidence intervals for the point estimates.

^i^Cannell, Brad, Livingston, Melvin, Burnett, Jason, Parayil, Megin, Reingle Gonzalez, Jennifer,M. Evaluation of the Detection of Elder Mistreatment through Emergency Care Technicians Project Screening Tool. 2020.

^j^Kue, Ricky, Ramstrom, Edward, Weisberg, Stacy, Restuccia, Marc. Evaluation of an emergency medical servicesbased social services referral program for elderly patients. 2009.

^k^Cannell, Brad, Gonzalez, Jennifer,M.Reingle, Livingston, Melvin, Jetelina, Katelyn,K., Burnett, Jason, Weitlauf, Julie,C. Pilot testing the detection of elder abuse through emergency care technicians (DETECT) screening tool: results from the DETECT pilot project. 2019.

^l^Nowak, K, Ouellette, L, Chassee, T, Seamon, J, Jones, J. Emergency Services Response to Elderly Abuse - Then and Now. 2018.

^m^Rinker, Austin G. Recognition and perception of elder abuse by prehospital and hospital-based care providers.2009.

^n^Nusbaum, N. J., Cheung, V. M., Cohen, J., Keca, M., Mailey, B. Role of first responders in detecting and evaluating elders at risk.2006.

^o^Namboodri, Brooke L., Rosen, Tony, Dayaa, Joseph A., Bischof, Jason J., Ramadan, Nadeem, Patel, Mehul D., Grover, Joseph, Brice, Jane H., Platts-Mills, Timothy F. Elder Abuse Identification in the Prehospital Setting: An Examination of State Emergency Medical Services Protocols. 2018.

#### Qualitative Analysis

Ten sources of evidence were included in qualitative analysis (Table 4) (Cannell et al., 2016; Cannell, Livingston, et al., 2020; Cimino-Fiallos & Rosen, 2021; Friese & Collopy, 2010; Gironda et al., 2010; Gonzalez et al., 2016; Rosen et al., 2017; Rosen et al., 2018; Salminen-Tuomaala et al., 2021; UN, 2002). Of the GRADE CerQual (nd) certainty of evidence screening for qualitative evidence, all outcomes were rated as demonstrating low or moderate levels of confidence due to concerns including, but not limited to, methodological limitations, coherence, adequacy, and relevance. Despite this, all ten sources provided valuable narrative insight that enriched the depth of analysis and provided nuanced understanding of the role of paramedics in responding to the vulnerabilities of older adults in abusive contexts. As such, the sources of evidence remained eligible for inclusion, to assist in shaping the findings and recommendations presented in the paper. Full GRADE CerQual (nd) certainty of evidence explanations are listed in Table 4 and Appendixes 3.2. One study, Mercier et al. (2020), did not undergo certainty of evidence assessment due to the unavailability of validated certainty of evidence tools or software for scoping reviews.

**Table 4.** CERQaul Summary of Findings Table (Qualitative Analysis).

#	Summarized review finding	GRADE-CERQual Assessment of confidence	Explanation of GRADE-CERQual Assessment	References
1	Improving prehospital detection of abuse of older adults requires a multi-faceted approach, including enhanced collaboration between emergency medical services, other professionals and system support to overcome organizational barriers integrating elderly abuse.	Low confidence	Moderate concerns regarding methodological limitations, Minor concerns regarding coherence, Moderate concerns regarding adequacy, and Moderate concerns regarding relevance	Gironda et al., 2010; Salminen-Tuomaala et al., 2021; United Nations, 2002; Rosen et al., 2018; Cimino-Fiallos & Rosen 2021; Gonzalez et al., 2016
2	Abuse of older adults is difficult to detect and identify during short patient interactions. Improving the initial training and continuing professional development curricula could improve identification of abuse of older adults, and response.	Moderate confidence	Moderate concerns regarding methodological limitations, Moderate concerns regarding coherence, Moderate concerns regarding adequacy, and Moderate concerns regarding relevance	Gironda et al., 2010; Salminen-Tuomaala et al., 2021; Friese & Collopy 2010; Rosen et al., 2018; Cimino-Fiallos & Rosen 2021; Rosen et al., 2017; Gonzalez et al., 2016; Cannell et al., 2020.
3	A user-friendly prehospital reporting tool is needed to increase confidence and efficiency in identifying and reporting potential cases of abuse of older adults.	Moderate confidence	Minor concerns regarding methodological limitations, Minor concerns regarding coherence, Moderate concerns regarding adequacy, and Minor concerns regarding relevance	Salminen-Tuomaala et al., 2021; Cimino-Fiallos & Rosen 2021; Rosen et al., 2017; Cannell et al., 2016; Gonzalez et al., 2016; Cannell et al., 2020

#### Narrative Summary of Findings

The synthesis below summarizes the stage in the review where extracted data was combined and evaluated. This determined the outcome of the review. The authors used textual descriptions to give expression to the qualitative data synthesis (Royal Melbourne Institute of Technology University, 2025). Although sub-headings are presented, the authors chose not to embark upon thematic or content analysis, due to the low and moderate confidence appraisals (Thomas & Harden, 2008). The synthesis aimed to show the outcomes and effects of various studies and identify methodological and quality concerns. The authors explicate the overall level of evidence, the degree of consistency of the findings, the effects of interventions and the associations found.

#### The Opportunity for Paramedics to Address Vulnerabilities of Older Adults in abusive contexts

Older adults are four times more likely to be transported to ED by ambulance than younger adults (Cannell et al., 2016; Nowak et al., 2018; Rosen et al., 2017). As the only healthcare providers who routinely enter the homes of health care consumers and are often the first medically trained personnel to evaluate ill and injured elderly patients, paramedics have a responsibility for identifying and responding to signs and symptoms of abuse and neglect among older adults (Rinker, 2009). When treating an older adult for illness or injury within their home, paramedics are also uniquely positioned to identify those who may be the victims of mistreatment, as these events may be the only time isolated and vulnerable older adults have contact with healthcare providers, or leave their homes (Rosen et al., 2017).

Additionally, most older adults who have experienced physical abuse or neglect present to the emergency department via ambulance, with 80% presenting with injuries sustained on the same day (Mercier et al., 2020). While abusers can be anyone who has a relationship with the older adult, research suggests that they are almost always a family member with whom the victim has a dependence relationship, in their home (Friese & Collopy, 2010). In residential care settings, abuse perpetration can be extended to staff, vendors or others (Friese & Collopy, 2010).

#### Clinical Case-Finding, Screening, and Reporting of Abuse and Neglect of Older Adults

Of emergency physicians and other allied care professionals, paramedics have the highest reporting rate of abuse of older adults to law enforcement authorities (Mercier et al., 2020). As the primary healthcare contacts of older adults experiencing abuse, paramedics should treat abuse and neglect as a physical disease, much like they screen and treat cardiac conditions, removing the veil of secrecy and increasing awareness of the ongoing epidemic (Rinker, 2009). With abuse, exploitation and neglect taking many forms, paramedics should observe and document inappropriate interactions between patients and their family or caregivers, the physical conditions and behavior of patients, the safety of home environments (including upkeep, safety concerns, sanitation, medical history and medication availability), and their professional instincts (Cannell et al., 2016; Cannell, Mars, & Schoen, 2020; Rosen et al., 2017). The secondary head-to-toe patient assessment is a key opportunity for paramedics to observe signs of abuse, and provide a comprehensive synthesis of signs, symptoms, clues, and patterns of different types that paramedics should be aware of, alongside documentation and reporting requirements (Friese & Collopy, 2010).

Despite the role of paramedics to report suspected cases of abuse against older adults, literature identified that over the past 20 years, the reasons for paramedics failing to report include unclear definitions of abuse, unawareness of mandatory reporting requirements, lack of reporting anonymity and lack of understanding of where reports should be made. These have remained consistently unchanged barriers to reporting (Nowak et al., 2018), notwithstanding increased knowledge about protection from potential litigation (based on good faith and the absence of malicious intent) if the reported case is determined to be unfounded (Nowak et al., 2018). Additional barriers to reporting cases of abuse were identified as a lack of existing protocols and training, communication issues, time constraints, and an absence of feedback on outcomes of reports made (Cannell, Mars, & Schoen, 2020). When screening tools, such as the “Detection of Elder Abuse Through Emergency Care Technicians” tool (DETECT), are made available in the out-of-hospital environment, monthly reports of abuse of older adults to appropriate authorities increased by over 220% (Mercier et al., 2020). Notwithstanding, this is not a consistently implemented screening tool across jurisdictions and countries. Studies also highlighted that changing the attitude and perspectives of paramedics towards abuse and neglect of older adults is key to ensuring abuse of older adults does not go underreported (Rinker, 2009). Alternative suggestions for ensuring paramedics meet their role of identifying and reporting abuse of older adults includes engaging the expanding role of community paramedicine to capitalize on the (early detection) skill set of paramedics and expand these programs to target interventions to ensure the safety of vulnerable older adults (Rosen et al., 2017). Clinical case-finding, selective or mandatory screening and clear reporting guidelines in health (and criminal justice) systems are paramount considerations in Paramedicine’s duty of care to older adults.

#### The Value of Multidisciplinary Collaboration to Leverage Responses to Abuse of Older Adults

To leverage the perspectives and competencies of all health professionals involved in the care of older adults, a multidisciplinary approach is crucial (Mercier et al., 2020). Multidisciplinary collaboration between health professionals may increase attention provided to at-risk groups, such as older adults, and consequently increase identification of common indicators of abuse (Salminen-Tuomaala et al., 2021). While paramedics can successfully screen older adults for mental health, environmental, and social problems including abuse or neglect, and refer them to social service agencies, they have reported difficulties in effectively conveying their concerns to emergency department (ED) staff (Rosen et al., 2018). While some barriers to this communication are due to time constraints, ED staff are often unavailable or not receptive to paramedic’s findings (Rosen et al., 2018). Literature recommends that, when possible, ED staff should seek out paramedics and enquire further about the home environment (scene survey), and impressions of the older adult patient, as well as review paramedic documentation and clinical observations (Rosen et al., 2018). Paramedics also have a unique opportunity to connect older adults with, or refer to, social services, which proves especially important for older adults who decline transport to hospital (Kue et al., 2009). This aligns with the United Nation’s recommendations to encourage health and social service professionals, as well as the public, to report suspected abuse of older adults, and inform older adults of the protection and support available to them (UN, 2002). There is also a further need for cooperation between government and civil or non-government organizations in addressing the abuse of older adults, as well as developing community initiatives (UN, 2002). This may include training in the detection of abuse of older adults in the curricular and continuing professional development of emergency care providers such as paramedics, nurses, and social workers (Salminen-Tuomaala et al., 2021).

## Discussion

The vulnerability of older adults is not limited to age alone, but rather the confounding factors of advancing age, co-morbidities, chronic medication compliance, and special needs that advance their overall frailty (Fernandez et al., 2002). Attending to older adults at their homes provides paramedics with the opportunity to detect and respond to cases of abuse and neglect. While paramedics have been identified as having the highest reporting rate (among allied health) of abuse of older adults to law enforcement authorities and are the primary healthcare contacts for older adults experiencing abuse (Mercier et al., 2020), this was a single finding in a single study. This finding is not necessarily generalizable to other EMS jurisdictions and countries. The higher reporting is likely to be a function of a disproportionately higher frequency probability of older adults who have experienced any form of abuse accessing emergency care in the acute setting, compared to access to other allied health professions. Notwithstanding, it is unknown if the reporting is optimal. The false positive cases, clinical and criminal justice outcomes and overall impact are unknown, as is the consistency of practice, screening practices, autonomy protection or coercive practices by paramedics, direct and vicarious trauma risk to practitioners, etc.

The evidence demonstrates the paramedic duty of care, their capacity for screening, detection and referral, and the value of interdisciplinary responses. The transformative value for the health care consumer is that they are deserving of and should receive consistent quality and evidence-based responses from the paramedic community. The dearth of evidence and the low quality of evidence suggests either undocumented or poorly defined practice. The transformative potential for paramedicine is that the need to professionalize responses and scientifically document approaches and impacts has been codified by the evidence within the review. This is a critical first step to defining the problem space or status quo in paramedic practice. Moving front-line workers from the fringe of the issue (or from unintended collusion) to violence disruptors enhances their value proposition as epidemiological sentinels and crisis interventionists. Paramedic interventions are usually *post-facto*, that is after the violence causes injury or harm. The value proposition or transformative potential of paramedicine in early detection (secondary prevention) and primary prevention warrants scholarly investigation and documentation (Tilley et al., 2024).

Consequently, to ensure a consistently responsive health system design, paramedics must continue to be trained and empowered to identify, respond to, and report cases of abuse and neglect against older adults. Friese and Collopy (2010) believe that simply handing a brochure about abuse of older adults to all patients over the age of 65 could be a method for paramedics to raise awareness about abuse and neglect. However, with 15% of older Australians, for example, experiencing a form of abuse within the last year (Australian Institute of Health and Welfare, 2024), more *must* be done. All paramedics should receive specific training for the detection of abuse of older adults (Gonzalez et al., 2016; Nowak et al., 2018; Rinker, 2009). For example, physical patterns of abuse of older adults are reportedly like that of children, with most injuries located on the upper limbs and maxillofacial regions (Mercier et al., 2020). Older females are also more likely to experience physical abuse and/or neglect, alongside older adults with higher levels of dependency on caregivers (Mercier et al., 2020; UN, 2002). Detailed clinical information, including physical signs and observations, alongside management and reporting plans should be included in ongoing paramedic education and sentinel surveillance to appraise the protective value of paramedic interventions.

While a pilot study of the DETECT screening tool demonstrated increased reporting rates, the tool’s overall acceptance and impact on patient outcomes are currently unknown (Mercier et al., 2020). There is currently a lack of reporting procedures integrated within paramedic protocols, with one study identifying that in the US, only 40% of state-wide paramedic protocols included information concerning abuse of older adults (Mercier et al., 2020) despite almost every (US) state having some form of mandatory reporting which is intended to protect older adults against abuse or neglect (Kayser et al., 2021). Despite paramedics demonstrating competence in reporting abuse and neglect of older adults (Mercier et al., 2020), there is a clear need for the development of an improved, evidence-informed screening or clinical case-finding tool that demonstrates efficacy in improving both reporting rates and patient outcomes, hence ensuring comprehensive protection for this vulnerable group. It is also evident that advancement of interprofessional collaboration between paramedics, emergency department (ED) staff, social services, and other allied health professionals would benefit the longitudinal care of at-risk older adults (Kue et al., 2009; Mercier et al., 2020; Rosen et al., 2017, 2018).

### Recommendations

The findings promote ambulance service performance (in relation to equity, effectiveness and efficiency in health care specific to older adults) and paramedic purpose and practices that can redress the complex prehospital needs of older adults before (primary prevention), during (early detection) or after (tertiary care) abusive contexts (Australian Government Productivity Commission, 2025). Following evidence synthesis, the authors have inferred several key evidence-based recommendations for enhancing the capacity of paramedics responding to older adults in abusive contexts or at risk there-of.

Development of an evidence-informed prehospital screening tool for detection and reporting of abuse and neglect of older adults must become a care system and clinical priority to enable early detection through clinical case-finding, selective or universal screening, relative to context. The tool should be based on the observations of the first responder, be easily incorporated into standard patient assessments, and provide paramedics with clear and simple reporting guidelines, adapted to their local jurisdiction and reporting requirements (Cannell et al., 2016, 2019; Mercier et al., 2020). This may progress to incorporating abuse and neglect screening of older adults into routine paramedic screening protocols and clinical guidelines (Cimino-Fiallos & Rosen, 2021; Namboodri et al., 2018) and may include development of an electronic reporting system that can automatically flag potential abuse cases and generate reports for social services (Gonzalez et al., 2016). Facilitation of ongoing research into the development and validation of efficient screening tools for identifying abuse and neglect of older adults in the prehospital environment must also be a priority (Namboodri et al., 2018).

Increase training of all paramedics on their role and responsibility to report suspected neglect and abuse of older adults (Cimino-Fiallos & Rosen, 2021; Gonzalez et al., 2016; Nowak et al., 2018; Rinker, 2009; Salminen-Tuomaala et al., 2021). Training should be mandatory but flexible, evidence-based, and include key abuse indicators including physical patterns of abuse, communication strategies for interacting with older adults who may be victim-survivors of abuse and/or neglect, safety resources and/or management plans, and reporting guidelines specific to local jurisdictional requirements (Gironda et al., 2010; Mercier et al., 2020).

A whole-of-health approach is needed to improve communication and collaboration between paramedics and/or prehospital health care providers, ED staff, and allied health including social services and/or adult protective services to ensure timely assessment and intervention in cases of suspected abuse and/or neglect (Mercier et al., 2020; Rosen et al., 2017, 2018). This can include implementation of a closed-loop system to facilitate referrals *and* follow-up with other healthcare providers following paramedic encounters with older adults (Kue et al., 2009). It may also involve utilizing the emerging field of community paramedicine to provide additional support and monitoring of older adults experiencing vulnerability (Rosen et al., 2018).

### Limitations

There is a scarcity of evidence on the paramedics’ role in addressing the needs or unmet needs of older adults within abusive contexts. Consequently, a specific search was conducted to minimize irrelevant sources, and thus some potential studies or gray literature may have been excluded due to a narrow search, notwithstanding hand searches and searches through gray literature databases. Studies appearing after the search period (such as Naidoo et al., 2024) may be of value, but fall outside this review. Studies were included from a variety of international sources, where the roles and responsibilities of paramedics may vary greatly depending on jurisdictional and legal guidelines. Therefore, some findings and recommendations may not apply to all ambulance settings. Inclusion criteria limited the search to articles written in English to reduce the risk of bias associated with translation, however, this may have resulted in missing relevant non-English works and cultural nuance. Included articles were limited to evidence published after 2000 due to both a limitation of time resources, and to ensure inclusion of contemporary practices and policies. This approach also ensured the inclusion of methodologically robust research considering the significant legislative and policy reform in health and security, abuse prevention and response, criminal justice, and the growing older adult population over the past 25 years. The review also did not find meta-analysis or in-depth quantitative analysis. While a risk of bias assessment was undertaken, lower quality data included within the study may decrease the strength of the findings. Additionally, one study (Mercier et al., 2020) did not undergo certainty of evidence assessment due to the unavailability of validated certainty of evidence tools or software for scoping reviews. The authors acknowledge that low levels of confidence from certainty of evidence screening may impact the generalizability and reliability of the findings. Finally, the findings seem to paint a paternalistic stance invoked by the disempowering nature of abusive contexts and, perhaps due to publication and other bias, does not equitably address older adults *with* capacity, competency, resources and resilience to care for and protect themselves and others from abusive contexts. This unintended de facto deficit approach that has the potential to reify stereotypes about older adults is acknowledged.

## Conclusion

The authors undertook a systematic review to determine the role of paramedics in addressing the vulnerabilities of older adults within abusive contexts. The review recognized that while there is a broad role for paramedics within these settings that includes clinical case-finding, screening, detection, referral and advocacy there is a paucity of direct research and context-specific evidence to guide their interventions and highlight best practice. Effective emergency planning and mandatory, comprehensive training on the specific needs of older adults is essential for paramedics, alongside developing evidence-informed guidelines and screening tools for risk and vulnerability. Further research, within ethical constraints, needs to be conducted to inform implementation across various jurisdictions. Doing so will likely improve ambulance service performance, increase the capacity of paramedics, at scale, in safely addressing the vulnerabilities of older adults within abusive contexts, thereby enhancing their health, safety and protection from preventable harm.

## Supplemental Material

Supplemental Material - Paramedic Roles, Purpose, and Practices when Responding to Older Adults in Abusive Contexts: A Systematic Review Supplemental Material for Paramedic Roles, Purpose, and Practices when Responding to Older Adults in Abusive Contexts: A Systematic Review by Megan De Silva, Aidan Peters, Benjamin De Waal, Lloyd Christopher, and Navindhra Naidoo in Journal of Applied Gerontology

## Data Availability

No original data is published, all data is publicly available in referenced results. Search strategies are publicly available in Appendix 1.

## References

[bibr1-07334648251330347] Australian Government Productivity Commission . (2025). Report on government services 2025: Part E, section 11 ambulance services. https://www.pc.gov.au/ongoing/report-on-government-services/2025/health/ambulance-services

[bibr2-07334648251330347] Australian Institute of Health and Welfare . (2019). Elder abuse: Context, concepts and challenges. Australian Government. https://www.aihw.gov.au/getmedia/affc65d3-22fd-41a9-9564-6d42e948e195/australias-welfare-chapter-7-summary-18sept2019.pdf.aspx

[bibr3-07334648251330347] Australian Institute of Health and Welfare . (2024). Family, domestic and sexual violence: Older people. Australian Government. https://www.aihw.gov.au/family-domestic-and-sexual-violence/population-groups/older-people

[bibr4-07334648251330347] Barker Th H. N. Aromataris E. Stone J. C. Leonardi-Bee J. Sears K. Hasanoff S. Klugar M. Tufanaru C. Moola S. Munn Z. (2024). The revised JBI critical appraisal tool for the assessment of risk of bias quasi-experimental studies. JBI Evidence Synthesis, 22(3), 378–388. 38287725 10.11124/JBIES-23-00268

[bibr5-07334648251330347] Bond M. C. Butler K. H. (2013). Elder abuse and neglect: Definitions, epidemiology, and approaches to emergency department screening. Clinics in Geriatric Medicine, 29(1), 257–273. 10.1016/j.cger.2012.09.004 23177610

[bibr6-07334648251330347] Campbell Jm K. M. Ding S. Carmody D. P. Hakonsen S. J. Jadotte Y. T. White S. Munn Z. (2020). Chapter 9: Diagnostic test accuracy systematic reviews. JBI Manual for Evidence Synthesis JBI. 10.1097/XEB.0000000000000061 26355602

[bibr7-07334648251330347] Cannell B. Gonzalez J. M. R. Livingston M. Jetelina K. K. Burnett J. Weitlauf J. C. (2019). Pilot testing the detection of elder abuse through emergency care technicians (DETECT) screening tool: Results from the DETECT pilot project. Journal of Elder Abuse & Neglect, 31(2), 129–145. 10.1080/08946566.2018.1564104 30614399

[bibr8-07334648251330347] Cannell B. Livingston M. Burnett J. Parayil M. Reingle Gonzalez J. M. (2020). Evaluation of the detection of elder mistreatment through emergency care technicians project screening tool. JAMA Network Open, 3(5), e204099. 10.1001/jamanetworkopen.2020.4099 32379330 PMC7206507

[bibr9-07334648251330347] Cannell B. Mars L. Schoen J. (2020). EAGLE and DETECT--innovative tools helping first responders to combat elder abuse. Generations, 44(1), 44–50. https://maltraitancedesaines.com/en/auteurs/cannell/

[bibr10-07334648251330347] Cannell M. B. Jetelina K. K. Zavadsky M. Gonzalez J. M. R. (2016). Towards the development of a screening tool to enhance the detection of elder abuse and neglect by emergency medical technicians (EMTs): A qualitative study. BMC Emergency Medicine, 16(1), 19. 10.1186/s12873-016-0084-3 27250247 PMC4888496

[bibr11-07334648251330347] Cimino-Fiallos N. Rosen T. (2021). Elder abuse-A guide to diagnosis and management in the emergency department. Emergency Medical Clinics Of North America, 39(2), 405–417. 10.1016/j.emc.2021.01.009 33863468

[bibr12-07334648251330347] Dahoud S. Simpson P. Naidoo N. (2024). Influence of patient sex on pain management practices in paramedicine: A rapid review. Paramedicine, 21(4), 168–180. 10.1177/27536386241240286

[bibr13-07334648251330347] Fernandez L. S. Byard D. Lin C. C. Benson S. Barbera J. A. (2002). Frail elderly as disaster victims: Emergency management strategies. Prehospital and Disaster Medicine, 17(2), 67–74. 10.1017/s1049023x00000200 12500729

[bibr14-07334648251330347] Friese G. Collopy K. T. (2010). Geriatric abuse. EMS Magazine, 39(7), 59–64. https://search.ebscohost.com/login.aspx?direct=true&db=rzh&AN=105052706&lang=en-gb&site=ehost-live 20687418

[bibr15-07334648251330347] Gironda M. W. Lefever K. Delagrammatikas L. Nerenberg L. Roth R. Chen E. A. Northington K. R. (2010). Education and training of mandated reporters: Innovative models, overcoming challenges, and lessons learned. Journal of Elder Abuse & Neglect, 22(3-4), 340–364. 10.1080/08946566.2010.490188 20711920

[bibr16-07334648251330347] GRADE-CERQual Interactive Summary of Qualitative Findings (iSoQ) . Norwegian Institute of public health (developed by the epistemonikos foundation, megan wainwright consulting and the Norwegian Institute of public health for the GRADE-CERQual project group). https://www.Isoq.epistemonikos.org

[bibr17-07334648251330347] Gradepro G. D. T. (2024). GRADEpro guideline development tool. McMaster University and Evidence Prime. https://www.Gradepro.org

[bibr18-07334648251330347] Joanna Briggs Institute . (2024). Aromataris E L. C. Porritt K. Pilla B. Jordan Z. (Eds.), JBI manual for evidence synthesis. Joanna Briggs Institute. 10.46658/JBIMES-24-01

[bibr19-07334648251330347] Kayser J. Morrow-Howell N. Rosen T. E. Skees S. Doering M. Clark S. Hurka-Richardson K. Bin Shams R. Ringer T. Hwang U. Platts-Mills T. F. Network T. G. (2021). Research priorities for elder abuse screening and intervention: A geriatric emergency care applied research (gear) network scoping review and consensus statement. Journal of Elder Abuse & Neglect, 33(2), 123–144. 10.1080/08946566.2021.1904313 33797344 PMC8204570

[bibr20-07334648251330347] Kue R. Ramstrom E. Weisberg S. Restuccia M. (2009). Evaluation of an emergency medical services-based social services referral program for elderly patients. Prehospital Emergency Care, 13(3), 273–279. 10.1080/10903120802706179 19499461

[bibr21-07334648251330347] Lefebvre C. Glanville J. Briscoe S. Littlewood A. Marshall C. Metzendorf M.-I. Noel-Storr A. Rader T. Shokraneh F. Thomas J. (2021). Technical supplement to chapter 4: Searching for and selecting studies. In Higgins J. P. T. Thomas J. Chandler J. Cumpston M. Li T. Page M. J. VA W. (Eds.) Cochrane handbook for systematic reviews of interventions. Cochrane. https://www.training.cochrane.org/handbook

[bibr22-07334648251330347] Lockwood C. M. Z. Munn Z. Porritt K. (2015). Qualitative research synthesis: Methodological guidance for systematic reviewers utilizing meta-aggregation. International Journal of Evidence-Based Healthcare, 13(3), 179–187. 10.1097/XEB.0000000000000062 26262565

[bibr23-07334648251330347] McArthur A K. J. Yan H. Florescu S. (2020). Chapter 4: Systematic reviews of text and opinion. In Aromataris E M. Z. (Ed.), JBI manual for evidence synthesis. JBI. https://10.0.182.66/JBIMES-20-05

[bibr24-07334648251330347] Mercier É. Nadeau A. Brousseau A.-A. Émond M. Lowthian J. Berthelot S. Costa A. P. Mowbray F. Melady D. Yadav K. Nickel C. Cameron P. A. (2020). Elder abuse in the out-of-hospital and emergency department settings: A scoping review. Annals of Emergency Medicine, 75(2), 181–191. 10.1016/j.annemergmed.2019.12.011 31959308

[bibr25-07334648251330347] Muka T. Glisic M. Milic J. Verhoog S. Bohlius J. Bramer W. Chowdhury R. Franco O. H. (2020). A 24-step guide on how to design, conduct, and successfully publish a systematic review and meta-analysis in medical research. European Journal of Epidemiology, 35(1), 49–60. 10.1007/s10654-019-00576-5 31720912

[bibr26-07334648251330347] Munn Z. Aromataris E. Barker T. H. Moola S. Tufanaru C. Stern C. McArthur A. Stephenson M. (2020). Methodological quality of case series studies: An introduction to the JBI critical appraisal tool. JBI Evidence Synthesis, 18(10), 2127–2133. 10.11124/JBISRIR-D-19-00099 33038125

[bibr27-07334648251330347] Munn Z. M. S. Lisy K. Riitano D. Tufanaru C. (2020). Chapter 5: Systematic reviews of prevalence and incidence. In Aromataris E M. Z. (Ed.), JBI manual for evidence synthesis. JBI. 10.46658/JBIMES-20-06

[bibr28-07334648251330347] Naidoo N. Feldman P. Muoio R. Sawyer S. Brijnath B. (2024). Strengthening the frontline response to elder abuse: Qualitative insights from Australian paramedics. Journal of Applied Gerontology, Article, 7334648241302455. 10.1177/07334648241302455 PMC12227804 39560298

[bibr29-07334648251330347] Naidoo N. Maria S. Flanagan B. Van Noordenburg A. Ho V. Mansour V. (2024). Paramedicine educators’ identity needs and impediments to professional emergence: A multiphase mixed-methods participatory approach. Journal of University Teaching and Learning Practice, 21(10), 1607. 10.53761/fw4g1607

[bibr30-07334648251330347] Namboodri B. L. Rosen T. Dayaa J. A. Bischof J. J. Ramadan N. Patel M. D. Grover J. Brice J. H. Platts-Mills T. F. (2018). Elder abuse identification in the prehospital setting: An examination of state emergency medical services protocols. Journal of the American Geriatrics Society, 66(5), 962–968. 10.1111/jgs.15329 29566428 PMC5992078

[bibr31-07334648251330347] National Ageing Research Institute Research . Retrieved 21 March 2024 from. https://www.nari.net.au/Pages/Category/research?Take=52

[bibr32-07334648251330347] Nowak K. Ouellette L. Chassee T. Seamon J. P. Jones J. (2018). Emergency services response to elder abuse and neglect - then and now. The American Journal of Emergency Medicine, 36(10), 1916–1917. 10.1016/j.ajem.2018.02.036 29510912

[bibr33-07334648251330347] Nusbaum N. J. Cheung V. M. Cohen J. Keca M. Mailey B. (2006). Role of first responders in detecting and evaluating elders at risk. Archives of Gerontology and Geriatrics, 43(3), 361–367. 10.1016/j.archger.2006.01.001 16513192

[bibr34-07334648251330347] Paez A. (2017). Gray literature: An important resource in systematic reviews. Journal of Evidence Based Medicine, 10(3), 233–240. 10.1111/jebm.12266 28857505

[bibr35-07334648251330347] Page M. J. McKenzie J. E. Bossuyt P. M. Boutron I. Hoffmann T. C. Mulrow C. D. Shamseer L. Tetzlaff J. M. Akl E. A. Brennan S. E. Chou R. Glanville J. Grimshaw J. M. Hróbjartsson A. Lalu M. M. Li T. Loder E. W. Mayo-Wilson E. McDonald S. Moher D. (2021). The PRISMA 2020 statement: An updated guideline for reporting systematic reviews. BMJ, 372(3), n71. 10.1136/bmj.n71 33782057 PMC8005924

[bibr36-07334648251330347] Reingle Gonzalez J. M. Cannell M. B. Jetelina K. K. Radpour S. Reingle Gonzalez J. M. (2016). Barriers in detecting elder abuse among emergency medical technicians. BMC Emergency Medicine, 16(1), 36–38. 10.1186/s12873-016-0100-7 27590310 PMC5010700

[bibr37-07334648251330347] Rethlefsen M. L. Kirtley S. Waffenschmidt S. Ayala A. P. Moher D. Page M. J. Koffel J. B. Brigham T. Chang S. Clark J. Conway A. Couban R. de Kock S. Farrah K. Fehrmann P. Foster M. Fowler S. A. Glanville J. PRISMA-S Group (2021). PRISMA-S: An extension to the PRISMA statement for reporting literature searches in systematic reviews. Systematic Reviews, 10(1), 39. 10.1186/s13643-020-01542-z 33499930 PMC7839230

[bibr38-07334648251330347] Rinker A. G. Jr. (2009). Recognition and perception of elder abuse by prehospital and hospital-based care providers. Archives of Gerontology and Geriatrics, 48(1), 110–115. 10.1016/j.archger.2007.11.002 18160115

[bibr39-07334648251330347] Rosen T. Lien C. Stern M. E. Bloemen E. M. Mysliwiec R. McCarthy T. J. Clark S. Mulcare M. R. Ribaudo D. S. Lachs M. S. Pillemer K. Flomenbaum N. E. (2017). Emergency medical services perspectives on identifying and reporting victims of elder abuse, neglect, and self-neglect. Journal of Emergency Medicine, 53(4), 573–582. 10.1016/j.jemermed.2017.04.021 28712685 PMC5660658

[bibr40-07334648251330347] Rosen T. Stern M. E. Elman A. Mulcare M. R. (2018). Identifying and initiating intervention for elder abuse and neglect in the emergency department. Clinics in Geriatric Medicine, 34(3), 435–451. 10.1016/j.cger.2018.04.007 30031426 PMC6057151

[bibr41-07334648251330347] Royal Melbourne Institute of Technology University . (2025). Teaching and research guides: Systematic reviews. RMIT University. Retrieved February 4 from. https://rmit.libguides.com/systematicreviews

[bibr42-07334648251330347] Salminen-Tuomaala M. Tiainen J. Mikkola R. Paavilainen E. (2021). Identification of elder abuse through out-of-hospital emergency care providers. Research and Theory for Nursing Practice, 35(3), 289–304. 10.1891/RTNP-D-20-00074 34140415

[bibr43-07334648251330347] Thomas J. Harden A. (2008). Methods for the thematic synthesis of qualitative research in systematic reviews. BMC Medical Research Methodology, 8(1), 45. 10.1186/1471-2288-8-45 18616818 PMC2478656

[bibr44-07334648251330347] Tilley D. Christopher L. D. Farrar T. Naidoo N. (2024). Emergency medical service responses as latent social capital toward deliberate self-harm, suicidality and suicide. Psychology Health & Medicine, 29(4), 743–753. 10.1080/13548506.2023.2214867 37200110

[bibr45-07334648251330347] United Nations . (2002). Report of the second World assembly on ageing. https://documents.un.org/access.nsf/get?OpenAgent&DS=A/CONF.197/9&Lang=E

[bibr46-07334648251330347] United Nations . Dag hammarskjöld library - resources & collections. United Nations. Retrieved 21 March 2024 from. https://www.un.org/en/library

[bibr47-07334648251330347] Veritas Health Innovation . (2024). Covidence systematic review software. https://www.covidence.org/

[bibr48-07334648251330347] Whiteman P. J. Macias-Konstantopoulos W. L. Relan P. Knopov A. Ranney M. L. Riviello R. J. (2023). Violence and abuse: A pandemic within a pandemic. The Western Journal of Emergency Medicine, 24(4), 743–750. 10.5811/westjem.58405 37527378 PMC10393453

[bibr49-07334648251330347] World Health Organization . WHO library and digital information networks. World Health Organization. https://www.who.int/library

