# Supplementary material for: Paramedic Roles, Purpose, and Practices When Responding to Older Adults in Abusive Contexts: A Systematic Review
Source: J Appl Gerontol. 2025 Apr 2;45(1):110–21. doi: 10.1177/07334648251330347 (PMC12681365; doi:10.1177/07334648251330347)
Supplement: Supplemental Material - Paramedic Roles, Purpose, and Practices when Responding to Older Adults in Abusive Contexts: A Systematic Review [file sj-pdf-1-jag-10.1177_07334648251330347.pdf]

APPENDIX 1 – Search Strings

**Population**	Paramedics and first responders, and older adults (defined as people over the age of 65 years)
Paramedic* OR ambulance* OR Emergency Medical Technician* OR Air Ambulance* OR emergency medical service* OR ems OR emt* OR pre?hospital OR first responder* OR emergency service* OR HEMS OR out?of?hospital' OR emergency technician* OR emergency practitioner* OR emergency d?spatch* OR emergency rescue* OR emergency resus* OR advanced life support OR community support co?ordinator OR allied health personnel	
AND	
elderly OR elder* OR old* person OR Nursing home* OR gerontologic care OR residential care OR residential facility OR aged care OR assisted living OR retirement facility OR retirement living OR 65 year* OR over 65	
**Concept**	Paramedic identification and response to vulnerabilities
**Context**	Abusive contexts including, but not limited to, institutional abuse, parental abuse, or gerontologic abuse.
abusive contexts OR institutional abuse OR parental abuse OR gerontologic abuse OR elder abuse	

APPENDIX 2 – Risk of Bias Assessment

JBI Critical Appraisal Checklist for Textual Evidence: Policy / Consensus Guidelines

**Study**	**Are the developers of the policy/ consensus guideline (and any allegiences/affiliations) clearly identified?**	**Do the developers of the policy/ consensus guideline have standing in the field of expertise?**	**Are appropriate stakeholders involved in developing the policy/guideline and do the conclusions drawn represent the views of their intended users?**	**Are biases due to competing interests acknowledged and responded to?**	**Are the processes of gathering and summarizing the evidence described?**	**Is any incongruence with the extant literature/evidence logically defended?**	**Are the methods used to develop recommendations described?**	**Inclusion?<br><br>Overall rating /7**
United Nations,	Yes	Yes	Yes	Unclear	Yes	N/A	Yes	Yes<br>5/7

2002								
Cimino-Fiallos & Rosen, 2021	Yes	Yes	Yes	No	No	N/A	Yes	Yes 4/7

JBI Critical Appraisal Checklist for Diagnostic Test Accuracy Studies

**Study**	**Was a consecutive or random sample of patients enrolled?**	**Was a case control design avoided?**	**Did the study avoid inappropriate exclusions?**	**Were the index test results interpreted without knowledge of the results of the reference standard?**	**If a threshold was used, was it pre-specified?**	**Is the reference standard likely to correctly classify the target condition?**	**Were the reference standard results interpreted without knowledge of the results of the index test?**	**Was there an appropriate interval between index test and reference standard?**	**Did all patients receive the same reference standard?**	**Were all patients included in the analysis**	**Inclusion? Overall rating /10**
Cannell, Livingston, et al. 2020	Yes	Yes	Yes	Yes	Yes	Yes	Yes	Yes	Yes	Yes	Yes 10/10

JBI Critical Appraisal Checklist for Qualitative Research

**Study**	**Is there congruity between the stated philosophical perspective and the research methodology?**	**Is there congruity between the research methodology and the research question or objectives?**	**Is there congruity between the research methodology and the methods used to collect data?**	**Is there congruity between the research methodology and the representation and analysis of data?**	**Is there congruity between the research methodology and the interpretation of results?**	**Is there a statement locating the researcher culturally or theoretically?**	**Is the influence of the researcher on the research, and vice-versa, addressed?**	**Are participants, and their voices, adequately represented?**	**Is the research ethical according to current criteria or, for recent studies, and is there evidence of ethical approval by**	**Do the conclusions drawn in the research report flow from the analysis, or interpretation, of the data?**	**Inclusion? Overall rating /10**

									an appropriate body?		
Cannell et al. 2016	Yes	Yes	Yes	Yes	Yes	Yes	Yes	Yes	Yes	Yes	Yes 10/10
Gonzalez et al. 2016	Yes	Yes	Yes	Yes	Yes	Yes	Yes	Yes	Yes	Yes	Yes 10/10
Mercier et al., 2020	Yes	Yes	Yes	Yes	Yes	Yes	Yes	Yes	Yes	Yes	Yes 10/10
Gironda et al., 2010	Yes	Yes	Yes	Yes	Yes	Yes	No	Yes	Yes	Yes	Yes 9/10
Rosen et al., 2017	Yes	Yes	Yes	Yes	Yes	Yes	No	Yes	Yes	Yes	Yes 9/10
Nowak et al., 2018	Yes	Yes	Yes	Yes	Yes	No	Yes	Yes	No	Yes	Yes 8/10
Rinker, 2009	Yes	Yes	Yes	Yes	Yes	No	No	Yes	No	Yes	Yes 7/10
Rosen et al., 2018	Yes	Yes	Yes	Yes	Yes	Yes	No	Yes	Yes	Yes	Yes 9/10
Salminen-Tuomaala et al., 2021	Yes	Yes	Yes	Yes	Yes	Yes	Yes	Yes	Yes	Yes	Yes 10/10

JBI Critical Appraisal Checklist for Case Series

**Study**	**Were there clear criteria for**	**Was the condition measured**	**Were valid methods used for**	**Did the case series have**	**Did the case series have**	**Was there clear reporting**	**Was there clear reporting**	**Were the outcomes or follow**	**Was there clear reporting**	**Was statistical analysis**	**Inclusion?**

	inclusion in the case series?	in a standard, reliable way for all participants included in the case series?	identification of the condition for all participants included in the case series?	consecutive inclusion of participants?	complete inclusion of participants?	of the demographics of the participants in the study?	of clinical information of the participants?	up results of cases clearly reported?	of the presenting site(s)/clinic(s) demographic information?	appropriate?	Overall rating /10
Kue et al., 2009	Yes	Yes	Yes	Yes	Yes	Yes	Yes	Yes	Yes	Yes	Yes 10/10

JBI Critical Appraisal Checklist for Quasi-Experimental Studies

Study	Is it clear in the study what is the "cause" and what is the "effect"?	Was there a control group?	Were participants included in any comparisons similar?	Were participants included in any comparisons receiving similar treatment/care, other than the exposure or intervention of interest?	Were there multiple measurements of the outcome, both pre and post the intervention /exposure	Were the outcomes of participants included in any comparisons measured in the same way?	Were outcomes measured in a reliable way?	Was follow-up complete and if not, were differences between groups in terms of their follow-up adequately described and analysed?	Was appropriate statistical analysis used?	Inclusion? Overall rating /10
Nusbaum et al., 2006	Yes	No	Unclear	N/A	No	N/A	No	Yes	Yes	Yes 4/7

JBI Critical Appraisal Checklist for Prevalence Studies

Study	Was the sample frame appropriate to address the target population?	Were study participants sampled in an appropriate way?	Was the sample size adequate?	Were the study subjects and the setting described in detail?	Was the data analysis conducted with sufficient coverage of the identified sample?	Were valid methods used for the identification of the condition?	Was the condition measured in a standard, reliable way for all participants?	Was there appropriate statistical analysis?	Was the response rate adequate, and if not, was the low response rate managed appropriately?	Inclusion? Overall rating /9
Cannell et al., 2019	Yes	Yes	Yes	Yes	Yes	Yes	Yes	Yes	Yes	Yes 9/9
Nambodri et al., 2018	Yes	No	No	Yes	Yes	Yes	Yes	Yes	No	Yes 6/9

JBI Critical Appraisal Checklist for Textual Evidence: Expert Opinion

Study	Is the source of the opinion clearly identified?	Does the source of opinion have standing in the field of expertise?	Are the interests of the relevant population the central focus of the opinion?	Does the opinion demonstrate a logically defended argument to support the conclusions drawn?	Is there reference to the extant literature?	Is any incongruence with the literature/sources logically defended?	Inclusion? Overall rating /6
Cannell, Mars, et al., 2020	Yes	Yes	Yes	Yes	Yes	Yes	Yes 6/6
Friese & Collopy, 2010	Yes	Yes	Yes	Yes	Yes	N/A	Yes 5/6

Appendix 3 – Certainty of Evidence Results

APPENDIX 3.1 – Certainty of Evidence: Evidence Profile Table (GRADEPro) / Quantitative Studies

Certainty assessment							Impact	Certainty	Importance
№ of studies	Study design	Risk of bias	Inconsistency	Indirectness	Imprecision	Other considerations			
**EMS Referral Rate for Suspected Elderly Abuse**									
2	non-randomised studies	serious[a]	not serious	not serious	not serious	none	The referral rate from EMS for suspected elderly abuse ranges from 0.5% to 4% of all reported cases of elderly abuse. [1,2]	⊕◯◯◯ Very low[a]	IMPORTANT
**Effectiveness of screening tools in increasing suspected abuse reporting and rates**									
2	non-randomised studies	very serious[b]	not serious	not serious	not serious	none	Implementation of the DETECT Screening tool increased the reporting rate by 3-fold (RR 3.03, CI 0.95: 2.06 - 4.46) in one study. In a second study the implementation of the same tool increased reporting more than two-fold (increased by 226%, p = 0.0056). [1,3]	⊕◯◯◯ Very low[b]	IMPORTANT
**Use of mandatory screening requirements for increasing screening rate**									
1	non-randomised studies	serious[c]	not serious	not serious	not serious	strong association	Changing from a system of voluntary screening to mandatory screening using a validation rule on ePCRs increased the screening rate from 5% (n = 25/549) to 86% (1222/1418). [3]	⊕⊕◯◯ Low[c]	IMPORTANT
**Accuracy of Prehospital Screening Tools for Elderly Abuse**									
2	non-randomised studies	very serious[d]	serious[d]	not serious	not serious	none	In one study screening accuracy using the DETECT tool was reported at 82% (95% CI: 77% to 87%) as verified by post screening case investigation. In another study (DETECT screening tool pilot study), the screening detection rate was 10% confirmed through matching screening records with subsequent case investigations. [1,3]	⊕◯◯◯ Very low[d]	IMPORTANT
**EMS Self-Reported Exposure to Elderly Abuse**									
3	non-randomised studies	very serious[e]	not serious	not serious	not serious	none	Between 58% to 65% of survey respondents indicated that they had encountered at least one case of suspected abuse in the past 6 months. In one study 64.3% of participants reported encountering at least 1 suspected case in the past 12 months.<br>One study (Nowak et al, 2018) estimated the mean number fo reported cases at 1.7 ± 5.0 per month and 18.1 ± 35.6 over the course of a career [4,5,6]	⊕◯◯◯ Very low[e]	IMPORTANT

EMS self reported screening practices, believes and attitudes towards screening

2	non-randomised studies	serious[e]	not serious	serious[f]	not serious	none	60.5% to 70% of participants indicated that they had not screened any patients for suspected elderly abuse in the past 12 or 6 months respectively.<br>In one study, Rinker et al. (2009) 89% of respondents indicated they where aware of their obligation to report, but 76% indicated that they did not believe that elderly abuse is a medical problem, with 20% indicating it is a social problem. In the same study 96.5% of particiants indicated that they did not believe elderly abuse was a rare event. [5,6]	⨁◯◯◯<br>Very low[e,f]	IMPORTANT

Formalised EMS Protocols for Elderly Abuse Screening and Reporting

1	non-randomised studies	very serious[g]	not serious	not serious	very serious[h]	none	40% (n = 14/35) of included states (USA) had formalised EMS protocols addressing elderly abuse. A total of 17.1% (n = 6/35) provide definitions for elderly abuse, 29% (n = 10/35) describe indicators for abuse, 22.8% (n = 8/35) provide guidelines for management and 34.3% (n = 12/35) provide instructions on reporting suspected abuse cases. [7]	⨁◯◯◯<br>Very low[g,h]	IMPORTANT

CI: confidence interval

Explanations

a. For one study (Cannell, 2020) this data was reported but was not part of the objectives of the study.
b. The study did not measure confirmed cases of abuse. It only matched screening reports to reports of investigations.
c. This study used a quasi-experimental design.
d. One of the studies (Cannell, 2019) was a pilot study. The authors retrospectively matched positive screening records with subsequent investigation records. As such there is no confirmation for false positive or false negative screening outcomes. As such the authors did not report on more formal measures of diagnostics accuracy.
e. The included survey studies used convenience samples of participants and self reported practice.
f. One of the studies included both hospital based and prehospital based providers.
g. This study only included publicly available protocols which could be found on public facing web interfaces.
h. Sample size limitations, wide confidence intervals for the point estimates.

References

1.Cannell, Brad, Livingston, Melvin, Burnett, Jason, Parayil, Megin, Reingle Gonzalez, Jennifer,M.. Evaluation of the Detection of Elder Mistreatment through Emergency Care Technicians Project Screening Tool.2020.
2.Kue, Ricky, Ramstrom, Edward, Weisberg, Stacy, Restuccia, Marc. Evaluation of an emergency medical servicesbased social services referral program for elderly patients.2009.
3.Cannell, Brad, Gonzalez, Jennifer,M.Reingle, Livingston, Melvin, Jetelina, Katelyn,K., Burnett, Jason, Weitlauf, Julie,C.. Pilot testing the detection of elder abuse through emergency care technicians (DETECT) screening tool: results from the DETECT pilot project.2019.
4.Nowak, K, Ouellette, L, Chassee, T, Seamon, J, Jones, J. Emergency Services Response to Elderly Abuse - Then and Now.2018.
5.Rinker, Austin G.. Recognition and perception of elder abuse by prehospital and hospital-based care providers.2009.
6.Nusbaum, N. J., Cheung, V. M., Cohen, J., Keca, M., Mailey, B.. Role of first responders in detecting and evaluating elders at risk.2006.
7.Namboodri, Brooke L., Rosen, Tony, Dayaa, Joseph A., Bischof, Jason J., Ramadan, Nadeem, Patel, Mehul D., Grover, Joseph, Brice, Jane H., Platts-Mills, Timothy F.. Elder Abuse Identification in the Prehospital Setting: An Examination of State Emergency Medical Services Protocols.2018.

Appendix 3.2 – Certainty of Evidence: Qualitative Studies (GRADE CERQual) / Qualitative Studies

#	Summarized review finding	Methodological limitations	Coherence	Adequacy	Relevance	GRADE-CERQual assessment of	References

						confidence	
1	Improving prehospital detection of abuse of older adults requires a multi-faceted approach, including enhanced collaboration between emergency medical services, other professionals and system support to overcome organisational barriers integrating elderly abuse.	Moderate concerns<br><br>**Explanation:** Moderate concerns regarding methodological limitations as the methods are poorly explained in two studies.	Minor concerns<br><br>**Explanation:** Minor concerns regarding coherence because the findings relate to EMT detection and reporting of Elder Abuse.	Moderate concerns<br><br>**Explanation:** Moderate concerns regarding adequacy because the participants in these studies represent a very small section of the emergency care profession.	Moderate concerns<br><br>**Explanation:** Moderate concerns regarding relevance because the findings are limited to two settings with well-developed EMS systems.	Low confidence<br><br>**Explanation:** Moderate concerns regarding methodological limitations, Minor concerns regarding coherence, Moderate concerns regarding adequacy, and Moderate concerns regarding relevance	Cimino-Fiallos & Rosen 2021; Gironda et al. 2010; Gonzalez et al. 2016; Rosen et al. 2018; SalminenTuomaala et al. 2021; United Nations 2002.
2	Abuse of older adults is difficult to detect and identify during short patient interactions. Improving the initial training and continuing professional development curricula could improve identification of abuse of older adults, and response.	Moderate concerns<br><br>**Explanation:** Moderate concerns regarding methodological limitations because two studies did not describe the research methods	Moderate concerns<br><br>**Explanation:** There are moderate concerns regarding coherence because one study focused on emergency department staff and the other on curriculum.	Moderate concerns<br><br>**Explanation:** Moderate concerns regarding adequacy because two studies do not discuss the research methods	Moderate concerns<br><br>**Explanation:** There are moderate concerns regarding relevance because one study focused on the emergency department. The other is on curriculum development.	Moderate confidence<br><br>**Explanation:** Moderate concerns regarding methodological limitations, Moderate concerns regarding coherence, Moderate concerns regarding adequacy, and Moderate concerns regarding relevance	Cannell et al. 2020; Cimino-Fiallos & Rosen 2021; Friese & Collopy 2010; Gironda et al. 2010; Gonzalez et al. 2016; Rosen et al. 2017; Rosen et al. 2018; SalminenTuomaala et al. 2021.
3	A user-friendly prehospital reporting	Minor concerns	Minor concerns	Moderate concerns	Minor concerns	Moderate confidence	Cannell et al. 2016; Cannell et al. 2020;

	tool is needed to increase confidence and efficiency in identifying and reporting potential cases of abuse of older adults.	**Explanation:** Minor concerns regarding methodological limitations because the methods used in the Rosen & Cimino-Fiallos are not described.	**Explanation:** Minor concerns regarding coherence because one study focused on the emergency department staff with EMS being considered as other health workers.	**Explanation:** Moderate concerns regarding adequacy because in only one study the focus was only on EMS	**Explanation:** Minor concerns regarding relevance because the findings are consistent with other research findings on this topic.	**Explanation:** Minor concerns regarding methodological limitations, Minor concerns regarding coherence, Moderate concerns regarding adequacy, and Minor concerns regarding relevance	Cimino-Fiallos & Rosen 2021; Gonzalez et al. 2016; Rosen et al. 2017; SalminenTuomaala et al. 2021.

APPENDIX 4.1 – Findings / Characteristics of Included Studies

Author and year of publication	Country	Study methodology /design	Findings
**Nowak et al. 2018**	United States of America	Grey literature	· The study population was a convenience sample of prehospital personnel throughout one state in 2015. Respondents estimated that they evaluated an average of 12.7±11.0 elderly patients per week (range:1–56), and approximately 53% of their respective patient population was 65 years of age or older. Although the prevalence of elder abuse in their communities was described as “rather rare” by many (43%) respondents, 65% had seen a suspected case of elder abuse during the past 6 months. The mean number of cases of elder abuse seen or suspected by an individual respondent during the past 6 months was 1.7 ± 5.0; the number of cases during their career averaged 18.1 ± 35.6.<br><br>· Compared to a similar Michigan study in 1995, slightly more cases of abuse are now reported when suspected (36% vs 27%, $p < 0.01$)<br>· Many paramedics and EMTs lack complete understanding of their role in identifying the reporting elder abuse<br>· Reasons for not reporting have not changed over the last 20 years: 1) personnel are unsure which authorities take reports, 2) unclear definitions of abuse, 3) personnel are unaware of mandatory reporting laws, 4) lack of anonymity

			when reporting<br>· Guidelines for identifying and reporting elder mistreatment within each state and locality should be taught to relevant PCPs, and when possible, written protocols should be established
**Mercier et al. 2020**	United States of America	Scoping Review	· Paramedics had the highest reporting rate of elder abuse to law enforcement authorities.<br>· In the out-of-hospital setting, after the implementation of the DETECT tool, the number of monthly reports of suspected elder abuse to the proper authorities increased by 5.4 times (226%).<br>· Most identified victims of physical abuse or neglect presented to the ED by ambulance, with 80% of presentations being for an injury sustained the same day.<br>· No out-of-hospital or ED studies dedicated to primary prevention of elder abuse were identified.<br>· A multidisciplinary approach was suggested to leverage the perspectives and competencies of every health care professional involved. For instance, paramedics often enter a patient's residence, giving them unique insight into living arrangements and concerns that may arise from informal observation. This information can be used to guide clinical practice and plan for a safe transfer of care into the community.<br>· Lack of reporting procedures was also an important concern.<br>· Second, increasing awareness of clinicians is a key step toward improving the care of this population. Multiple studies have demonstrated the inadequate training and<br>insufficient knowledge relative to elder abuse that contribute to poor detection rates. Web sites dedicated to geriatric out-of-hospital and ED care and education, including basic knowledge and clinical pearls, can also be used to improve knowledge and deliver continuous medical education.<br>· There is a consensus among clinicians and researchers focusing on geriatric care that elder abuse is likely one of the most underrecognized and underreported conditions in the out-of-hospital and ED settings. Nevertheless, the out-of-hospital and ED environments are unique in that they provide an important opportunity to care for vulnerable populations.<br>· Abuse has been linked to long-term debilitating psychological effects, including anxiety, depression, and suicidal ideation, as well as increased risk of hospitalization, long-term facility placement, and death. Delay in recognizing elder abuse has a detrimental effect on the patient's quality of life and outcome. Accordingly, elder abuse has been identified as one of the most important geriatric research priorities.<br>· Factors associated with elder abuse:<br>- Female sex was more common in identified cases of neglect and physical abuse in ED literature.<br>- The level of dependence on the caregiver seemed to have an influence on the risk of being physically abused or neglected.<br>· Regarding physical findings associated with physical abuse, two thirds of the injuries were located on the upper extremities and the maxillofacial region.<br>· According to health care professionals, the lack of training for diagnosis, intervention, documentation, and reporting is likely to contribute to current low rates of recognition<br>· The DETECT tool is the only one developed for the out-of-hospital setting. It was shown to improve reporting rates in a pilot study, but its generalizability, acceptance, and effect on patient-important outcomes are unknown.
**Rinker 2009**	United States of	Descriptive Survey	· PCPs (including EMTs and paramedics) are the most common contacts for victims of elder abuse and neglect<br>· It is of utmost importance that PCPs can recognise the signs and symptoms of elder abuse and neglect

	America		· PCPs must treat elder abuse and neglect as a physical disease and screen our patients suffering from this condition as we screen those suffering from other medical conditions<br>· Elder abuse and neglect is an ongoing epidemic and the “veil of secrecy” needs to be removed<br>· Only a comprehensive training program covering all aspects of abuse and neglect can curve the epidemic<br>· Altering the personal attitudes of PCPs towards the elderly, abuse and neglect is also vital
**Rosen et al. 2017**	United States of America	Qualitative study	· Older adults (aged ≥65) are four times more likely than younger patients to utilize EMS services and represent 38% of total EMS responses with transport to the ED.<br>· Cases of elder abuse, neglect, and self-neglect are common in geriatric patients and may have serious medical consequences but are rarely identified. An estimated 5–10% of older adults experience elder mistreatment each year. This mistreatment may include physical abuse, sexual abuse, emotional/psychological abuse, financial exploitation, or neglect.<br>· Many older adults self-neglect, threatening their own health and safety by failing to perform or refusing assistance with essential self-care. Elder mistreatment victims have significantly increased mortality and are at higher risk for adverse health outcomes including depression, disability, hospitalization, and nursing home placement.<br>· Evaluation by health care providers for acute injury or illness represents an important potential opportunity to identify elder mistreatment, as this may be the only time these vulnerable and isolated older adults leave their home.<br>· EMS providers, who perform initial assessments of ill and injured patients after activation of the 911 system, often in a patient’s home, are uniquely positioned to identify vulnerable older adults who may be mistreatment victims. While providing acute care and transporting, EMS may observe unusual or inappropriate interactions between the caregiver or family and the patient. They may also observe or investigate the safety of the home environment for the older adult, including cleanliness and upkeep as well as the availability of food, medications, and heat.<br>· Many participants reported significant interest in adopting protocols to assist in protecting older adults, and suggested several specific strategies to overcome the barriers that prevent EMS providers from effectively reporting their findings. Participants identified seven solutions to improve communication and reporting of vulnerable older adults. These included the opportunity to photographically document the home living situation, additional training for EMS providers, improved direct communication with social workers, a dedicated location on existing forms or a new form to document concerns, a hotline for reporting, a system to provide feedback to EMS providers on outcomes of the cases they identify, and community paramedicine with home visits.<br>· EMS providers who conduct initial assessments of patients, often in their home environment, may play a critical role in identification, reporting, and intervention for vulnerable victims of elder mistreatment.<br>· EMS may be particularly helpful in the detection of self-neglect, which our participants reported seeing most commonly.<br>· Participants also suggested the potential for community paramedicine to intervene and ensure the safety for vulnerable older adults. This is an intriguing way to capitalize on EMS providers’ unique skill set and role within the community. Using community paramedicine among older adults to improve health care utilization is already being evaluated, and expanding these programs to target vulnerable adults may help to provide services to those who need them most.
**Cannell et al. 2016**	United States of America	Qualitative study	· Older adults are four-times more likely to use in- home emergency medical services than younger adults. EMTs are uniquely positioned to identify potential abusive or neglectful situations.<br>· EMTs can identify indicators of abuse or neglect (such as family interactions, home upkeep, medication availability,

			safety concerns and sanitation) not available to other emergency personnel, social workers or health care providers<br>· Under-reporting is of particular concern because social isolation, dementia, and health and functional status are risk factors for elder abuse and may hinder older adults' ability to self-identify and self-report abuse or neglectful situations.<br>· The results of the current study provide evidence that EMTs do, in fact, regularly interact with older adults who are living with risk factors for abuse, and that they desire a screening tool to help them overcome current barriers to reporting<br>· Recommendations are development of an EMT screening tool of elder mistreatment that is brief and based on observable characteristics for the environment, patient, caregiver and context.
**Friese & Collopy. 2010**	United States of America	Expert opinion	· As EMS professionals, we are the rare healthcare providers who have access to patients' homes and can observe living conditions, interaction with caregivers and other clues to different types of abuse. In addition to providing immediate lifesaving interventions and stabilization, our other top responsibility is to document, and report known or suspected elder abuse.<br>· EMS professionals use the patient assessment to determine the nature and extent of injuries but can also use it to look for clues of elder abuse.<br>· EMS professionals are most likely to find clues of elder abuse while conducting the secondary assessment. Note any injuries that seem incongruous with the complaint and/or mechanism of injury found with the focused or detailed head-to-toe physical examination. Some potential signs of physical abuse include:<br>- Musculoskeletal injuries like sprains, strains, fractures and dislocations<br>- Bruises in unusual areas like the inner arm or inner thigh<br>- Burns from hot water or cigarettes<br>- Wounds (bruises, burns, abrasions) in various stages of healing<br>- Abrasions and/or bruises from being firmly held, pulled or restrained<br>- Recurring injuries to the same area of the face, neck or upper extremities<br>- Unusual markings from hand grips, bites, ropes or other restraints.<br>· Signs of sexual abuse might be noted as you conduct a physical examination or disrobe a patient for a procedure like 12-lead ECG tracing.<br>· EMS professionals may also observe suspected abusers.<br>· Your patient care report will be a valuable component in the investigation of elder abuse. An objective and comprehensive report that factually reports the assessment findings and treatments provided is critical.<br>· There is a role for EMS professionals in preventing elder abuse. Simply handing a brochure about elder abuse to all patients over 65 could be a simple way to raise awareness.
**Cannell, Livingston, et al. 2020**	United States of America	Expert opinion	· First responders are uniquely positioned to detect and document EM. Unlike other providers, emergency medical services and law enforcement are routinely and frequently called to older adults' residences and can observe older adults in the context of their living environments and caregiving situations. Access to observe cues in the physical and social environment creates opportunities to identify, report, and document EM.<br>· Many frontline workers think they lack the knowledge and skills to accurately recognise and report signs of EM and welcome tools that might help them to do so.<br>· The DETECT project is designed to help medics identify, document, and report instances of potential mistreatment and neglect occurring in the community.<br>· Medics reports that they frequently can observe characteristics of the older adult's physical and social environment that

			are indicators of potential EM.<br>· DETECT tool has led to a four-fold increase in medical reporting of elder mistreatment to APS.<br>· This research identified 4 other barriers to reporting commonly experienced by medics:<br>- Apprehension about infringing upon older adult's freedom to choose the conditions of their living environments<br>- Moral anxiety about the perceived negative consequences of an APS investigation<br>- Time burden associated with marking a report to adult protective services<br>- Perceived lack of case-recall ability<br>Limitations<br>- Researchers are not able to yet calculate any measure of diagnostic performance<br>Pilot studies were not designed to gather contextual information about EM cases, therefore missing the opportunity to under-stand the full breadth of physical and social indicators that may serve as early warning signs of EM
**Gonzalez et al. 2016**	United States of America	Qualitative study	· Social isolation, dementia, and health and functional status are risk factors for elder abuse and may hinder older adults' ability to self-identify and self-report abuse or neglectful situations.<br>· Older adults with cognitive or functional limitations may fear retaliation by a family member or caregiver, and as a result, continue to live in abusive or neglectful situations.<br>· Because older adults are four-times more likely to use in-home emergency medical services than younger adults, EMTs are uniquely positioned to identify potential abusive or neglectful situations.<br>· EMTs can identify indicators of abuse or neglect (such as family interactions, home upkeep, medication availability, safety concerns and sanitation) not available to other emergency personnel, social workers or health care providers.<br>· Findings suggest at least 5 barriers inhibiting EMT's ability to detect and/or report abuse or neglect.<br>1. Older adults may elect or even prefer to live in environments that EMTs perceive as intrinsically neglectful. This reduces the EMT's confidence in making the decision to report abuse.<br>2. The decision to report a suspected case to APS weighs heavily on EMTs, as they bear the moral burden of "wrecking someone's life" based upon "gut" instincts that abuse may be occurring. EMTs also highlighted the consequences associated with reports of suspected elder abuse to APS.<br>3. Time restrictions prevent EMTs from reporting all causes of abuse they encounter<br>4. At the end of a 12-hour shift, EMTs reported difficulty recalling sufficient information about a patient during a call to APS<br>5. The use of a checklist or brief screening tool that could automatically generate and transmit a report to APS would increase EMTs confidence in reporting potential cases of elder abuse or neglect to APS.<br>· Training on the indicators of abuse and neglect is needed.<br>· These consistent findings highlight the need for an integrated reporting system that could automatically flag potential abusive or neglectful situations and generate a report transmitted to APS without additional burden on the EMT.<br>· EMTs are eager to work with APS to address the under-reporting of elder abuse and neglect, but the current reporting procedures are time-prohibitive given their role as emergency healthcare providers.<br>· Currently, EMTs receive only thirty minutes or less of training on elder abuse and neglect during their basic certification course. Therefore, additional continuing education courses should be focused on training EMTs to identify potential elder abuse or neglect and communicate the details of these cases with APS.<br>· All EMTs agreed that a brief checklist would increase their confidence level in reporting to APS, and as a result, reduce

			their moral anxiety. These new protocols (e.g., training and checklists) could simplify and automate the processes associated with reporting suspected cases to APS, and as a result, link older adults in potentially dangerous situations with assistance.
**United Nations, 2002**	Spain	Policy	- Professionals need to recognize the risk of potential neglect, abuse or violence by formal and informal caregivers both in the home and in community and institutional settings.<br>- The process of ageing brings with it declining ability to heal, so that older victims of abuse may never fully recover physically or emotionally from trauma. The impact of trauma may be worsened because shame and fear cause reluctance to seek help.<br>- Older women face greater risk of physical and psychological abuse due to discriminatory societal attitudes and the non-realization of the human rights of women<br>· Elimination of all forms of neglect, abuse and violence of older persons.<br>Action:<br>- Sensitize professionals and educate the public, using media and other awareness-raising campaigns, about elder abuse and its various characteristics and causes<br>- Encourage cooperation between Government and civil society, including non-governmental organizations, in addressing elder abuse by, inter alia, developing community initiatives<br>- Minimize the risks to older women of all forms of neglect, abuse and violence by increasing public awareness of, and protecting older women from, such neglect, abuse and violence, especially in emergency situations<br>- Encourage further research into the causes, nature, extent, seriousness and consequences of all forms of violence against older women and men and widely disseminate findings of research and studies.<br>· Creation of support services to address elder abuse<br>Action:<br>- Encourage health and social service professionals as well as the public to report suspected elder abuse<br>- Encourage health and social service professionals to inform older persons suspected of suffering abuse of the protection and support that can be offered<br>Include handling of elder abuse in the training of the caring professions
**Cimino-Fiallos & Rosen. 2021**	United States of America	Text and opinion	· Older patients are four times more likely to use EMS services than younger adults, and EMS providers interact with these patients and their caregivers in their home. Given this rate of utilisation, innovative approaches to the diagnosis of elder abuse are needed to increase detection in the prehospital setting<br>· EMS providers, including paramedics, have the unique advantage of seeing inside a patient's home and can comment on habitability and availability of resources, such as food and medication<br>· These professions sometimes develop relationships with patients who frequently use their services and can report signs of physical decline or worsening living conditions<br>· Physicians in the ED should utilise EMS perspective when evaluating patients with other suggestions of abuse or neglect<br>· Ideally, screening protocols only target the individuals at the highest risk for abuse. Doing so would require fewer resources than universal screening and may improve specificity<br>· Having EMS providers screen for elder mistreatment as part of their routine protocols may increase detection of

			mistreatment
**Nusbaum et al. 2006**	United States of America	Quantitative: Survey	· First responders generally recognised that the elder could be placed at risk, not only by the actions of others, but also from their own actions<br>· Of the first responders surveyed, 58% estimated that in the last six-months they had met at least one older individual who might have been abused or neglected, and 77% estimated that in the last six-months they had met at least one older individual living in a situation where they posed a danger to themselves or others<br>- Only 2% of the responders indicated that in the last six-months they had screened more than 20 older individuals for abuse or neglect, and a similarly small percent of first responders indicated that they had screened more than 20 older individuals for poor judgement<br>· The data suggests that first responders are an important resource for identifying elders at risk. At the same time, the data suggests that formal screening for elder vulnerability is not a widespread practice among first responders<br>· There is considerable willingness to recognise that some older individuals should not drive a motor vehicle, but a far lower recognition of the risk to impaired elders from possession of a firearm<br>· First responders represent an important but underutilised resource to identify elders at risk
**Gironda et al. 2010**	United States of America	Text and opinion	· Paramedics have a pivotal role in abuse detection and response; however, it was apparent that many professionals did not have adequate basic information on normal aging by which to measure issues of neglect and abuse. Even mandated reporters needed accurate benchmarks for interpreting behaviour of elderly patients and caregivers<br>· There were two main challenges identified:<br>Challenge 1 – Engaging the reluctant trainee<br>Some of the projects found their target trainees to be reluctant participants, believing that learning about elder abuse and neglect was either not within their professional responsibility or was not a problem among their clients<br>Challenge 2 – Reaching busy professionals<br>Curriculm content can be presenting either synchronously or asynchronously. Each modality has characteristics that either enhance or detract from the training program depending on the expectations of the learners and the nature of the content<br>· The creation of a dedicated elder abuse and neglect training and resource Website requires extensive staff time and funding, but such sites are highly visible through search engines and provide universally accessible and up-to-date information and training. Sites such as this can continue to inform and motivate those who have been trained after formal sessions, as well as be a frontline resource for anyone searching for assistance or information regarding elder abuse and neglect.<br>· Features of future elder abuse and neglect training programs include the need to be flexible, able to be presented on multiple educational platforms, asynchronous (available any time the users need them or are available), self-contained, and continuously monitored for accuracy and currency.<br>· Future training programs will have modules ready to respond to opportunities for training as they arise, allowing for continuous revision that ensures appropriate, timely, and up-to-date content. Future training will also make use of online ability for interactive role playing and responding to relevant, real-world scenarios developed for the particularly professional target group
**Kue et al.**	United	Case series	· EMS provides an invaluable opportunity to connect the elderly with social services at the time of contact. EMS

**2009**	States of America		encounters with the elderly represent a unique opportunity for health care providers to assess both medical complaints and social concerns that may otherwise not be addressed, especially if a patient refuses transport to an ED<br>· Paramedics have the ability to accurately assess both the patient's critical condition, and the environmental context in which an injury or illness took place<br>· Providing paramedics with a closed-loop system would facilitate proper follow-up with other providers such as case managers, visiting nurses, and primary care physicians, who may otherwise never become aware of the current issue at hand<br>· The need for social service follow-up appeared to be more obvious to paramedics if presented with a patient complaint related directly to a non-medical complaint<br>· In this study, paramedics appeared to refer more social service related complaints compared with other categories such as fall assistance. This highlights a difference in perception of social service needs among paramedics and represents an area for further training and education
**Namboodri et al. 2018**	United States of America	Cross-sectional study	· Studies assessing EMS attitudes towards elder abuse found that, although EMS providers express a desire to identify and report elder abuse, they often feel that they have insufficient guidance regarding how to do so<br>· Of the 35 statewide protocols (EMS), 14 (40%) mention elder abuse, of which 43% define it. Of the 28 protocols (80%) that mention child abuse, 32% define it. The protocols that define elder abuse often clarify that abuse include physical, sexual, emotional, or neglectful acts or omissions by self or others, and/or the illegal use of person or property for profit or advantage. 10 of the 14 protocols that mention elder abuse list indicators of elder abuse (physical findings, psychosocial factors, and condition of the home environment – content is primarily focused on physical indicators e.g. Burns, fractures, bruises. Although these indicators are important, they are insufficient for providing a robust approach to identifying elder abuse. Physical abuse is less common than other forms of elder abuse such as neglect, so findings from a physical examination will capture only a small proportion of older adults experiencing abuse. Additional indicators that EMS might use to identify elder abuse include characteristics of the home environment such as broken utilities, lack of heating or cooling, presence of odours, an empty refrigerator, extreme clutter, expired medication, and safety hazards. In addition, consideration of caregiver behaviour, or the conspicuous absence of a caregiver, might provide further insights into the presence of elder abuse). 8 protocols that mention elder abuse also describe management of older adults suspected to be experiencing abuse, and 12 provide instructions regarding reporting.<br>· There is a need for more substantial protocols regarding elder abuse identification and intervention, clearer instructions regarding when these protocols should be implemented, dissemination of these protocols throughout the country, and more structured reporting guidelines and mechanisms<br>· Considering the unique position EMS providers are in to identify geriatric problems beyond elder abuse, improvements and dissemination of EMS protocols to identify elder abuse might overlap with efforts to identify older adults in need of additional support, such as a referral to a social worker or food assistance program.<br>· Given the high prevalence and substantial morbidity of elder abuse, elder abuse screening is an important additional focus for these EMS-led community programs – because of the large number of older adults transported and the limited time of EMS providers to screen, an optimal approach to elder abuse screening may be a multi-tier screen in which a simple, brief, but fairly sensitive initial set of observations is used to identify individuals who might be at risk, for additional assessment by EMS providers or ED nurses and physicians.<br>· Additional studies are needed to develop and test instruments that efficiently and accurately leverage the unique

			position of EMS providers to identify elder abuse in the pre-hospital setting
**Cannell, Mars, et al. 2019**	United States of America	Cohort study	· During the pilot period, 258 medics participated in an eligible 911 response. Of those 258 medics, 251 (97%) completed at least one DETECT screening. Prior to the validation rule, 19% of medics who participated in an eligible 911 response completed at least one DETECT screening, compared to 99% after the validation rule<br>· 21 of the 209 (10%) positive DETECT screenings were matched to completed APS investigations. 25 of 1038 (2%) negative DETECT screenings were matched to a completed APS investigation.<br>· Immediately following the introduction of the DETECT screening tool, there was an increase of 5.4 (p=0.0056) APS reports per month, a 226% improvement<br>· The most frequently completed item (99.9% of screenings) was number 1: unusual odour (eg. Urine, faeces) and the most frequently skilled items were 15 and 16 (if caregiver/s present, does the patient/older adult appear to lack social support or family that cares for them, and, if caregiver/s present, does the patient/older adult hesitate to talk with you or discuss their condition in front of the caregiver?)<br>· The item with the highest percentage of "yes" responses (8.7%) was item number 26, "Does the patient/older adult need assistance with eating, toileting, transferring, dressing, or bathing?". The item/s with the lowest percentage of a "yes" response were items number 14 and 16 (If caregiver/s present, they appear to be dependent on the patient/older adult for financial or other needs?, and, If caregiver/s present, does the patient/older adult hesitate to talk with you or discuss their condition in front of the caregiver/s?)<br>· Each of the 26 screening items was observed multiple times by medics practicing in the field. These are all positive indicators that support the feasibility of using the DETECT screening tool in practice. However, the DETECT screening tool produces a high rate of false positives<br>· The study's preliminary evidence of its utility for increasing detect and reporting of probable EM cases offers hope of a true advancement in the field of geriatric medicine and brings us a step closer to addressing the public health quandary of elder maltreatment
**Rosen et al. 2018**	United States of America	Text and opinion	· EMS can successfully screen older patients for mental health, environmental, and social problems including elder abuse and refer them to service agencies.<br>· EMS providers report difficulties effectively communication their concerns to ED providers because of barriers including time constrains and ED staff who are unavailable or not receptive. When possible, ED providers should proactively seek out prehospital personnel and inquire about their impression of the patient and the home environment, and EMS call report documentation should always be reviewed. Increasing use by EMS of electronic record accessible to ED providers may help to ensure that their observations are available to and used by ED providers
**Cannell et al. 2020**	United States of America	Prevalence study	· Medics within the MedStar service area reported more than 4 times as many cases of elder mistreatment during the implementation of DETECT<br>· The occurrence of elder mistreatment was validated in 83% (95%CI, 75-91%) of the reports investigated by APS in the periods when MedStar medics did not have access to the DETECT screening tool, compared with 82% (95% CI, 77-87%) in the periods when MedStar medics had access to the DETECT screening tool, indicating that there were no difficulties in the proportion of reports that resulted in a validated APS investigation. In other words, there was no

			evidence that the increases in reporting associated with the DETECT tool use were disproportionately invalid or inappropriate reports<br>· This study provides evidence that incorporating the DETECT screening tool into the routine practices of medics may be associated with substantial increases in the frequence in which they report potential cases of EM to APS. Of importance, there was no evidence that the observed increase in reporting frequency was accompanied by a decrease in the appropriateness of those reports.<br>· Use of the DETECT tool was associated with the investigation and intervention of elder mistreatment cases that may have otherwise gone unnoticed and unreported<br>· This finding suggests that incorporating the DETECT screening tool into the routine practice of medics is associated with substantial increases in the frequency in which medics report potential cases of elder mistreatment to APS
**Salminen-Tuomaala et al. 2021**	Finland	Qualitative research	· According to emergency care providers, the short duration of care contacts makes the identification of elder abuse challenging for them. They were seldom called to respond to any form of abuse. Instead, suspicion of abuse was commonly raised during the patient interview and examination.<br>· Despite the short contact, the interviewees felt that they, as first responders, had a frontline view of the identification of elder abuse. They said that it was possible to gain comprehensive information about the situation by means of patient interviews and observation of the home environment. Signs of physical abuse were the easiest to identify. A more comprehensive interview, study on context, and more time would be required for the detection of psychological, social, or financial abuse.<br>· The emergency care providers found that despite the short contacts, they were able to identify cases of elder abuse, as they were frequently the first healthcare professionals to see the clients following abusive incidents.<br>· According to the emergency care providers, it was impossible to gain an understanding of family dynamics and culture, and of the various forms of contact. The signs and consequences of neglected care, however, could be rapidly detected. The interviewees also found it relatively easy to identify concrete factors in the environment and living conditions that threatened the client's safety or coping<br>· The participants estimated that elder abuse had increased over the past few years and a great number of cases has been reported than earlier<br>· The participants agreed that the abusers were commonly family members, paid care providers, or persons otherwise close to the elders; they were seldom unfamiliar persons.<br>· The results support the international observation that the extent of elder abuse is sufficiently large for these professionals to encounter it on a daily basis.<br>· It is essential that professionals in emergency medical services report their observations as decreed by law<br>· Training on the detection of elder abuse should be included in nursing and social work curricular, and in the continuing professional development of emergency care providers.<br>· The identification of elder abuse may be improved by multiprofessional collaboration, increased attention given to risk groups, and common indicators of abuse, and by adoption of creation of screening tools to assist detection and reporting

Adult protective services (APS), Centre for Disease Control (CDC), confidence interval (CI), Detection of Elder Abuse Through Emergency Care Technicians tool (DETECT), electrocardiogram (ECG), emergency department (ED), elder mistreatment (EM), emergency medical service (EMS), emergency medical technicians (EMTs), pre-hospital care providers (PCPs).

Appendix 4.2 Characteristics of Included Quantitative Studies

Study	Country / Setting	Methodology	Participants	Question / Aim	Outcomes
**Cannell, Livingston, et al. 2020**	United States Texas, 45 cities in 3 counties.	Quasi Experimental (Difference – in-Differences Design)	**Experimental Group**: EMTs in Medstar Area (DETECT tool implemented) **Control Group Comparison**: EMTs outside Medstar service area (DETECT tool not implemented) & Non EMTs in Medstar service area	Investigate the association between use of the DETECT screening tool and number of elderly abuse reports made over 3 years	Reports to Adult Protection Services (APS). 11,178 adult patients included in the study (65 – 105 years). In total 18080 reports of elder mistreatment, neglect or exploitation, 4% (667) made by EMTs. EMTs in the DETECT tool arm reported 3 time more cases of elder mistreatment (Adjusted RR 3.03; 95% CI, 2.06 – 4.46). The accuracy of screening was reported at 83% (95% CI:75% to 91%) pre-DETECT implementation and 82% (95% CI: 77% to 87%) after DETECT implementation verified through case investigation by APS.
**Cannell et al., 2019**	United States Fort Worth, Texas	Pilot Study – Exact design not specified.	EMTs employed by Medstar Service (Texas)	Evaluate procedural barriers to using DETECT tool and documenting the rate of detection of individual screening indicators in the DETECT tool	A total of 1967 eligible patients included of which 1247 (63.3%) where screened. Use of the tool increase screening from 5% (25/549) to 86% (1222/1418) from before to after implementation. A total of 209 (16.8%) positive screenings reported of which 21 (10%) where matched with an APS investigation record while 188 (90%) did not have an APS investigation record. A total of 1038 (83%) negative screenings reported, with 25 (2.4%) subsequently found to have APS investigation reports. More than two-fold increase in reporting (226% improvement in reporting rate per month.
**Kue et al., 2009**	United States Single City in State of Massachusetts	Retrospective Case Series	EMS Providers & EMS Physicians.	Objective was to document EMS implementation of a senior support program within a city.<br><br>Document the frequency of referral of elder patient by EMS to the support program, including demographic	Included Adults > 60 years (referral program criterion). Of 6429 patients seen by EMS a total of 721 patients met inclusion criteria, 23 (3%) where referred by EMS (1% specifically for abuse) of which 7 (1%) were lost to follow-up and 1 declined referral. A total of 698 underwent retrospective chart review by EMS physicians. 47 (6.5%) were subsequently referred. 15 (2%) where lost to follow-up and 23 (3%) declined referral.<br>Overall referral rate = 70/721 (9%) for all causes (not only abuse) of which 24 accepted referral (34%). In 651 cases (90.2%) of cases investigators indicated they were unable to initiate referral. A total of 7 patient were referred specifically for abuse / neglect. (1%) with 4 of

				characteristics of patients and reason for referral	those being referred by EMS providers and 3 on chart review by EMS physicians. Patient enrolled by EMS where more likely to have a social service-related complaint (48% vs 15%, p = 0.005). EMS referrals where also associated to higher follow-up referral acceptance (94% vs 28%, p < 0.001)
**Nusbaum et al., 2006**	United States (One city)	Quasi-Experimental Before – After Study	First Responders including Police and Fire-fighters	Testing the effectiveness of an educational intervention to improve recognition of vulnerable elderly patients	Only one set of survey results reported. The reported results are of the pre-intervention survey.<br><br>Pre-Intervention Survey:<br>58% of respondents indicated that they had encountered a suspected elder abuse case in past 6 months.<br>70% indicated that they had not screened any elderly patients in the past 6 months for suspected abuse, with only 2% indication they had screened more than 20 cases.
**Namboodri et al., 2018**	United States (National)	Cross Sectional Descriptive Survey	Published Statewide EMS protocols for identification management and reporting of elderly abuse	Describe statewide EMS protocols for identification management and reporting of elderly abuse	35 Statewide protocols included. 14/35 (40%) mention elder abuse, 6/35 (17.1%) provide definitions, 10/35 (29.5%) describe indicators or abuse, 8/35 (22.8%) provide guidelines for management and 12/35 (34.3%) provide instruction on reporting.
**Rinker, 2009**	United States State of Maryland	Descriptive Survey	Convenience sample of Prehospital Care Providers & Hospital Care Providers<br>N = 400, response rate of 400/645 = 62%, 272/400, prehospital providers & 127/400, hospital-based providers	Exposure to elderly abuse & neglect.<br>Ability to recognize elderly abuse & neglect<br>Knowledge, beliefs and Attitudes towards elderly abuse & neglect	55% of prehospital respondents reported no exposure to elderly abuse or neglect in pass 12 months, 34% reporting seeing 1 to 3 patients, with 11% reporting seeing more > 4 patients, with 1% indicating they see > 20 such patients in the past 12 months<br>64.3% of prehospital providers reported identifying 1 or more patients in the last 12 months was living in circumstances where they were a danger to themselves or others.<br>60.5% of respondents indicated that in the past 12 months they had not screened any patients for elderly abuse. (both PCP and HCP)<br>89% of respondents indicated that they are aware of their obligation to report suspected abuse. (both PCP and HCP)<br>76% Indicated they believed elderly abuse not to be a medical issue – with 20% indicating they believe it is a social problem. (both PCP and HCP)<br><br>96.5% indicated that they believe that elderly abuse is not a rare event.

					(both PCP and HCP)<br>Other measurements include beliefs about which signs may be indicators of abuse and neglect.
**Nowak et al., 2018**	United States – One State (Michigan)	Descriptive Survey	Convenience sample of Prehospital EMS personnel, (n=155)	Determine exposure to, attitudes towards elder abuse and knowledge of mandatory reporting laws.<br>Secondary objective was to compare these results with a previous survey conducted in 1995.	43% of Respondents classified incidence of elder abuse as 'rather rare'.<br>A total of 65% of respondents indicated that they had encountered at least one case of suspected abuse in the past 6 months.<br>Mean number of self-reported cases over past 6 months by participant = 1.7 ± 5.0, and 18.1 ± 35.6 over the course of a career.

Appendix 4.3 Characteristics of included qualitative studies

Author(s), Year	Setting	Study Design	Data Collection Methods	Main Findings
**Cannell et al. 2016**	Participants (23) were recruited from a mobile healthcare provider in a metropolitan area and a regional office in North Texas, USA	Grounded theory was used as a qualitative method to research Elderly Abuse. Emergency Medical Technicians (EMTs) and Adult Protection Service (APS) caseworkers participated in five focus groups.	11 EMTs and 12 APS participated in five focus groups.<br>Data saturation was reached by the fifth focus group. The audio recording was transcribed, and two coders used the 'two rivers' to code and identify themes.	The study identified eight domains of items that might be included in a screening tool for EMTs. These domains are: (1) exterior home condition; (2) interior living conditions; (3) social support; (4) medical history; (5) caregiving quality; (6) the physical condition of the older adult; (7) older adult's behaviour; and (8) EMTs gut instincts. EMTs expressed a desire to work more closely with APS
**Gironda et al. 2010**	City College of San Francisco, USA	Informal research reported when the Archstone Foundation launched the Elder Abuse and Neglect Initiative to design a curriculum and deliver training for professionals (including EMTs and paramedics).	The Health Education and Community Health Studies Department at the City College of San Francisco developed a project team that used informal conversations to solicit information from firefighters and paramedics at the college.	Participants estimated that over 90% of the calls they respond to involve elders. Most paramedics and EMTs have encountered severe cases of neglect and<br>self-neglect. They were unaware of resources to address the situation and felt that little could be done or were afraid of making matters worse. Those who<br>worked for private companies were discouraged from reporting by their employers as it could jeopardise contracts. Some didn't realise that they<br>could report neglect or self-neglect. Some saw so many cases that

				they did not consider them out of the ordinary or reportable. Many reported<br>that they lacked the time to explore suspicions.
**Reingle Gonzalez et al. 2016**	Participants (23) were recruited from a mobile healthcare provider in a metropolitan area and a regional office in North Texas in the USA	Grounded theory was used as a qualitative method to research Elderly Abuse. Emergency Medical Technicians (EMTs) and Adult Protection Service (APS) caseworkers participated in five focus groups.	11 EMTs and 12 APS participated in five focus groups.<br>Data saturation was reached by the fifth focus group. The audio recording was transcribed, and two coders used the 'two rivers' to code and identify themes.	Findings suggest several barriers prevent EMTs from reporting elder abuse to APS. Participants suggested that they had limited training on elder abuse detection or reporting. EMTs suggested that training and creating an automated reporting system or a brief screening tool could enhance EMTs' ability to detect and communicate suspected elder abuse cases to APS.
**Rosen et al. 2017**	The article is based on data from studies conducted in the USA	Not specified as this is a guide to the di	Not specified	Emergency medical service (EMS) providers, including paramedics, have the unique advantage of seeing inside a patient's home and can comment on habitability and availability of resources, such as food and medication. Physicians should utilise the EMS perspective when evaluating patients with other suggestions of abuse or neglect.<br>Having EMS providers screen for elder mistreatment as part of their routine protocols may increase the detection of mistreatment.<br>The Detection of Elder Mistreatment Through Emergency Care Technicians screening tool incorporates EMS providers' observations of a patient's emotional state, living conditions, physical symptoms, and interactions with caregivers.
**Salminen-Tuomaala et al. 2021**	Twelve prehospital emergency care providers and community paramedics from a hospital district in Finland participated in the study	Inductive content analysis of interviews of participants	Individual theme interviews were held with the participants in 2019. The findings were summarised into meaning units and similar items grouped to form categories.	The identification of elder abuse may be improved by multi-professional collaboration. Training on the detection of elder abuse should be included in<br>curricula and in the continuing professional development of emergency care providers.

REFERENCES

Cannell, B., Gonzalez, J. M. R., Jetelina, K. K., Livingston, M., Burnett, J., & Weitlauf, J. C. (2019). Pilot testing the detection of elder abuse through emergency care technicians (DETECT) screening tool: results from the DETECT pilot project. *Journal of Elder Abuse & Neglect*, *31*(2), 129-145. https://doi.org/10.1080/08946566.2018.1564104

Cannell, B., Livingston, M., Burnett, J., Parayil, M., & Reingle Gonzalez, J. M. (2020). Evaluation of the Detection of Elder Mistreatment Through Emergency Care Technicians Project Screening Tool. *JAMA Network Open*, *3*(5), e204099-e204099. https://doi.org/10.1001/jamanetworkopen.2020.4099

Cannell, B., Mars, L., & Schoen, J. (2020). EAGLE and DETECT--Innovative Tools Helping First Responders to Combat Elder Abuse. *Generations*, *44*(1), 44-50. https://search.ebscohost.com/login.aspx?direct=true&db=rzh&AN=142988390&lang=en-gb&site=ehost-live

Cannell, M. B., Jetelina, K. K., Zavadsky, M., & Gonzalez, J. M. R. (2016). Towards the development of a screening tool to enhance the detection of elder abuse and neglect by emergency medical technicians (EMTs): a qualitative study. *BMC Emergency Medicine*, *16*, 1-10. https://doi.org/10.1186/s12873-016-0084-3

Cimino-Fiallos, N., & Rosen, T. (2021). Elder Abuse-A Guide to Diagnosis and Management in the Emergency Department. *Emerg Med Clin North Am*, *39*(2), 405-417. https://doi.org/10.1016/j.emc.2021.01.009

Friese, G., & Collopy, K. T. (2010). Geriatric abuse. *EMS Magazine*, *39*(7), 59-64. https://search.ebscohost.com/login.aspx?direct=true&db=rzh&AN=105052706&lang=en-gb&site=ehost-live

Gironda, M. W., Lefever, K., Delagrammatikas, L., Nerenberg, L., Roth, R., Chen, E. A., & Northington, K. R. (2010). Education and training of mandated reporters: innovative models, overcoming challenges, and lessons learned. *Journal of Elder Abuse & Neglect*, *22*(3/4), 340-364. https://doi.org/10.1080/08946566.2010.490188

Gonzalez, J. M. R., Cannell, M. B., Jetelina, K. K., Radpour, S., & Reingle Gonzalez, J. M. (2016). Barriers in detecting elder abuse among emergency medical technicians. *BMC Emergency Medicine*, *16*, 1-8. https://doi.org/10.1186/s12873-016-0100-7

Kue, R., Ramstrom, E., Weisberg, S., & Restuccia, M. (2009). Evaluation of an emergency medical services-based social services referral program for elderly patients. *Prehospital Emergency Care*, *13*(3), 273-279. https://doi.org/10.1080/10903120802706179

Mercier, É., Nadeau, A., Brousseau, A.-A., Émond, M., Lowthian, J., Berthelot, S., Costa, A. P., Mowbray, F., Melady, D., Yadav, K., Nickel, C., & Cameron, P. A. (2020). Elder Abuse in the Out-of-Hospital and Emergency Department Settings: A Scoping Review. *Annals of Emergency Medicine*, *75*(2), 181-191. https://doi.org/10.1016/j.annemergmed.2019.12.011

Namboodri, B. L., Rosen, T., Dayaa, J. A., Bischof, J. J., Ramadan, N., Patel, M. D., Grover, J., Brice, J. H., & Platts□Mills, T. F. (2018). Elder Abuse Identification in the Prehospital Setting: An Examination of State Emergency Medical Services Protocols. *Journal of the American Geriatrics Society*, *66*(5), 962-968. https://doi.org/10.1111/jgs.15329

Nations, U. (2002). *Report of the Second World Assembly on Ageing*.

Nowak, K., Ouellette, L., Chassee, T., Seamon, J. P., & Jones, J. (2018). Emergency services response to elder abuse and neglect - Then and now. *Am J Emerg Med*, *36*(10), 1916-1917. https://doi.org/10.1016/j.ajem.2018.02.036

Nusbaum, N. J., Cheung, V. M., Cohen, J., Keca, M., & Mailey, B. (2006). Role of first responders in detecting and evaluating elders at risk. *Archives of Gerontology & Geriatrics*, *43*(3), 361-367. https://doi.org/10.1016/j.archger.2006.01.001

Rinker, A. G., Jr. (2009). Recognition and perception of elder abuse by prehospital and hospital-based care providers. *Arch Gerontol Geriatr*, *48*(1), 110-115. https://doi.org/10.1016/j.archger.2007.11.002

Rosen, T., Stern, M. E., Elman, A., & Mulcare, M. R. (2018). Identifying and Initiating Intervention for Elder Abuse and Neglect in the Emergency Department. *Clinics in Geriatric Medicine*, *34*(3), 435-451. https://doi.org/10.1016/j.cger.2018.04.007

Rosen, T., Stern, M. E., McCarthy, T. J., Clark, S., Mulcare, M. R., Flomenbaum, N. E., Lien, C., Lachs, M. S., Bloemen, E. M., Mysliwiec, R., Ribaudo, D. S., & Pillemer, K. (2017). Emergency Medical Services Perspectives on Identifying and Reporting Victims of Elder Abuse, Neglect, and Self-Neglect. *Journal of Emergency Medicine (0736-4679)*, *53*(4), 573-582. https://doi.org/10.1016/j.jemermed.2017.04.021

Salminen-Tuomaala, M., Tiainen, J., Mikkola, R., & Paavilainen, E. (2021). Identification of Elder Abuse Through Out-of-Hospital Emergency Care Providers. *Research & Theory for Nursing Practice*, *35*(3), 289-304. https://doi.org/10.1891/RTNP-D-20-00074
